# Beyond lethal doses: sub-MIC levels of multi-class antibiotics induce species- and isolate-dependent biofilm inhibition in gram-negative pathogens

**DOI:** 10.1186/s12866-026-05620-5

**Published:** 2026-09-26

**Authors:** Raghda Elawady, Aliaa G. Aboulela, Ahmed N. Amer, Abeer A. Ghazal, Ahmed Gaballah

**Affiliations:** 1 Department of Microbiology, Medical Research Institute, Alexandria University, Alexandria, Egypt; 2 Department of Pharmaceutical Microbiology and Immunology, Faculty of Pharmacy and Drug Manufacturing, Pharos University, Alexandria University, Alexandria, Egypt

**Keywords:** Sub‑MIC, Biofilm modulation, Crystal violet, PCR, DNA abundance, Inhibition, Matrix

## Abstract

**Background:**

Biofilm-associated infections remain a major clinical challenge due to their intrinsic tolerance to antimicrobial therapy. Sub-minimum inhibitory concentrations (sub-MICs) of antibiotics are frequently encountered in vivo, particularly at sites of infection and on indwelling medical devices. However, their influence on biofilm formation is controversial, with evidence of both stimulatory and inhibitory effects. Clarifying these responses may reveal novel therapeutic opportunities for managing nosocomial biofilm-related infections.

This study aims to investigate the effects of three sub-MIC concentrations (25%, 50%, and 75% of MIC) of five antibiotic classes on biofilm formation in *Pseudomonas aeruginosa*, *Escherichia coli*, and *Klebsiella pneumoniae* using two distinct biofilm quantification methods.

**Materials and methods:**

In this study, the effects of 5 different antimicrobial agents, namely, azithromycin, gentamicin, ciprofloxacin, doxycycline, and imipenem, at different sub-MIC concentrations (12.5%, 25%, and 50% of MIC) were tested on 5 different clinical isolates of *P. aeruginosa*, *E. coli*, and *K. pneumoniae*. Clinical isolates were exposed to sub-MIC levels of antibiotics in a standardized biofilm assay, with statistical comparisons conducted to determine significant biofilm modulation using crystal violet (CV) staining to measure total biomass accumulation and quantitative PCR (qPCR) to assess qPCR-derived genome-equivalent DNA abundance within biofilms. qPCR detects DNA originating from viable, dead, damaged, or lysed cells, as well as extracellular DNA (eDNA).

**Results:**

Across the 75 tested conditions (5 antibiotics × 3 sub-MIC concentrations × 5 clinical isolates per species), sub-inhibitory antibiotic exposure produced pronounced, species- and target-dependent biofilm responsiveness varied by species: *P. aeruginosa* demonstrated the highest biomass modulation (62% CV significance), *E. coli* exhibited the greatest molecular responsiveness (55% qPCR significance), and *K. pneumoniae* showed lower overall modulation (47% CV, 32% qPCR significance). Imipenem exerted the highest biomass suppression in *P. aeruginosa* (80% CV vs. 13% qPCR inhibition), whereas doxycycline predominantly reduced genome equivalents in *P. aeruginosa* (73% qPCR vs. 7% CV inhibition). In *E. coli*, azithromycin produced peak biomass inhibition (73% CV vs. 7% qPCR inhibition), while gentamicin led to molecular suppression (60% qPCR vs. 27% CV inhibition). In *K. pneumoniae*, azithromycin and ciprofloxacin directed to biomass inhibition (both 40% CV), while azithromycin produced the highest qPCR reduction (33% qPCR). Bidirectional modulation was widespread across drug classes. Azithromycin acted as a primary inducer in *P. aeruginosa* (80% CV, 67% qPCR induction), while imipenem shifted from a biomass inhibitor in *P. aeruginosa* (80% CV inhibition) to a primary inducer in *K. pneumoniae* (47% CV, 60% qPCR induction). Unidirectional responses were drug- and method-specific, such as imipenem in *P. aeruginosa* CV (80% inhibition) and doxycycline in *P. aeruginosa* qPCR (73% inhibition).

**Conclusion:**

This study demonstrates that sub-MIC exposure produces heterogeneous, antibiotic-, species-, isolate-, concentration-, and method-dependent biofilm responses that diverge between structural matrix accumulation and genomic abundance. Therefore, our findings highlight that sub-MIC exposures demand a multi-assay diagnostic framework to accurately evaluate biofilm-associated drug responses.

**Supplementary Information:**

The online version contains supplementary material available at https://doi.org/10.1186/s12866-026-05620-5.

## Introduction

Biofilms are structured microbial communities encased in an extracellular matrix, which provides bacteria with enhanced survival advantages, including increased resistance to antimicrobial agents. Their presence in nosocomial infections, especially those associated with indwelling medical devices, poses significant challenges in clinical treatment. The restricted penetration of antibiotics into biofilms, combined with adaptive resistance mechanisms, contributes to persistent infections and therapeutic failures [1].

Antimicrobials fail to penetrate the whole biofilm, even if they can achieve the high concentration required to saturate the biofilm matrix. The hindered antimicrobial penetration can be due to many causes [2]. One of them is the probability of antimicrobial binding to the biofilm matrix components or adherence to the bacterial membrane itself [3, 4]. Although the restricted penetration is considered a temporary mechanism, it grants the biofilm cells adequate time to develop more tolerance [5].

A grave medical concern exists worldwide as a result of the widespread antimicrobial resistance and extensive use of antibiotics. Since they are frequently exposed to sub-MIC dosages of antimicrobials, biofilm-forming bacteria are given particular attention. In general, sub-MIC antibiotics may influence bacterial morphologies, pathogenicity, and biofilm development even when they don’t have any negative side effects [6, 7]. 

Numerous studies showed how pathogenic bacteria’s capacity to form biofilms was affected by exposure to low concentrations of antimicrobial agents. However, it is still conflicting and differs depending on the antimicrobial agent, bacterial strain, and biofilm detection method. Several studies have investigated the impact of sub-MIC levels on biofilm dynamics [8, 9]. However, conflicting findings underscore the need for comparative analysis across multiple bacterial species and antibiotic classes using diverse quantification techniques. Conventional crystal violet (CV) staining measures total biomass accumulation but does not distinguish between bacterial cell viability and matrix density. Conversely, qPCR provides a molecular assessment of bacterial cell count within biofilms, offering complementary insights into biofilm modulation.

This study aims to assess the effects of sub-MIC concentrations (50%, 25%, 12.5%) of five antibiotic classes (azithromycin, ciprofloxacin, doxycycline, gentamicin, and imipenem) on biofilm formation in three prominent Gram-negative bacteria (*Pseudomonas aeruginosa*,* E. coli*, and *K. pneumoniae*). Biofilm modulation was assessed using two complementary methods: CV staining to quantify total biomass and quantitative PCR (qPCR) to measure genome-equivalent DNA abundance within biofilms. qPCR detects DNA originating from viable, dead, damaged, or lysed cells, as well as eDNA. This dual-method approach enhances the reliability of biofilm quantification and provides a comprehensive understanding of whether antibiotic effects are confined to matrix production or extend to bacterial cell abundance.

## Materials and methods

### Bacterial isolates and minimum inhibitory concentration (MICs) of antimicrobial agents

This in vitro experimental study included 15 clinical isolates representing three Gram-negative species (*P. aeruginosa*, *E. coli*, and *K. pneumoniae*), along with one reference strain for each. Clinical isolates were obtained from patients admitted to the Medical Research Institute, Alexandria University between January 2023 and June 2024. Inclusion criteria required Gram-negative isolates (*P. aeruginosa*, *E. coli*, *K. pneumoniae*) recovered from clinically significant infections (respiratory, urinary, wound, bloodstream). Duplicate isolates from the same patient were excluded to ensure sample independence. Identification was performed using conventional biochemical tests (oxidase, indole, citrate, urease) and confirmed by the VITEK-2 automated system (bioMérieux, France). Antimicrobial susceptibility testing was conducted using broth microdilution in cation-adjusted Mueller-Hinton broth (CAMHB) in accordance with CLSI guidelines (2021), and isolate-specific MICs for ciprofloxacin, gentamicin, doxycycline, azithromycin, and imipenem are reported in Supplementary Table S1. Isolates were exposed to three sub-MIC levels (12.5%, 25%, and 50% of MIC) of five representative antibiotic classes: ciprofloxacin (fluoroquinolone), gentamicin (aminoglycoside), doxycycline (tetracycline), azithromycin (macrolide), and imipenem (carbapenem). Isolate-specific MIC values, clinical susceptibility interpretations (S/I/R), and specimen sources are comprehensively detailed in Supplementary Table S1.

All assays were performed in triplicate to ensure reproducibility. Antibiotics were obtained from certified suppliers (Sigma-Aldrich, AUG Pharma, El-Nile Co., Alexandria Co., European Egyptian Pharm. Ind.) with purity ≥ 98%. Stock solutions were prepared using sterile distilled water or appropriate solvents (e.g., DMSO for doxycycline) and filter-sterilized. Active concentrations were calculated based on molecular weight and potency provided by the manufacturer. Sub-MIC levels were expressed consistently as fractions of MIC (**½×**,** ¼×**, and **⅛×** MIC), corresponding to 50%, 25%, and 12.5% of the MIC value, respectively. For each isolate–antibiotic combination, the actual concentrations in µg/mL are reported in Supplementary Table S2. When MICs exceeded the maximum tested concentration (128 µg/mL), sub-MICs were calculated relative to this upper limit, and such cases are explicitly noted in the Supplementary Table. This approach ensured that all sub-MIC exposures were standardized and reproducible across isolates and antibiotic classes.

A Reference strains (*P. aeruginosa* ATCC 27853, *E. coli* ATCC 25922, and *K. pneumoniae* ATCC 700603) were used only as a methodology control in biofilm formation to validate the CV staining method quantification methodology. The MICs of 5 different classes of antimicrobial agents, ciprofloxacin (fluoroquinolones class, EUROPEAN EGYPTIAN PHARM. IND., Cairo, Egypt), gentamicin (aminoglycosides class, Alexandria Co. for pharmaceutical & clinical industries, Cairo, Egypt), doxycycline (tetracycline class, El-Nile Co. for pharmaceutical & clinical industries, Cairo, Egypt), azithromycin (macrolide class, AUG pharma, Cairo, Egypt), and imipenem (carbapenem class, Sigma-Aldrich, UK), was determined according to the Clinical and Laboratory Standards Institute (CLSI; 2021) guidelines for broth microdilution susceptibility testing [10]. Isolates were cultured on blood agar, standardized to 0.5 McFarland turbidity in Mueller-Hinton broth (MHB), and exposed to serial antibiotic dilutions (ranging from 0.06 to 128 µg/mL) to determine MIC endpoints.

### Biofilm Formation

Biofilm formation was assessed using two complementary quantification methods:

#### Detection of total biomass by CV staining

Biofilm assays were performed in 96-well plates with a final well volume of 100 µL (50 µL inoculum + 50 µL antibiotic or control medium). The final inoculum was adjusted to ~ 1 × 10⁶ CFU/mL after dilution of the initial 0.5 McFarland suspension to avoid excessively high starting densities. Wells were supplemented to a final glucose concentration of 0.5% to promote biofilm formation. Each condition was tested in triplicate wells (technical replicates) across five independent isolates per species (biological replicates). Controls included untreated biofilm wells and blank wells containing medium only. Biofilm formation was allowed to develop undisturbed at 37 °C for 24 h. After incubation, wells were washed three times with 200 µL PBS to remove planktonic cells, and background absorbance from blank wells was subtracted. The attached biofilm biomass was heat-fixed at 60 °C for 60 min before staining with 0.1% (w/v) CV solution. After a 15-minute incubation, excess stain was removed by washing, and absolute ethanol was used to resolubilize the bound dye. Optical density (OD) measurements were recorded at 620 nm using a microplate reader. Biofilm biomass was normalized to untreated controls using the formula: (OD₆₂₀ treated / OD₆₂₀ control) × 100. Heat fixation at 60 °C for 60 min was applied before CV staining to stabilize the biofilm matrix and minimize detachment during subsequent washing and staining steps. 

#### Quantitation of biofilm DNA using qPCR

Biofilms were prepared as described for CV staining. Cells were scraped, resuspended in 100 µL saline, boiled at 100 °C for 10 min, cooled on ice, and centrifuged at 15,000 g for 10 min consistent with published protocols for rapid debris removal without DNA loss [11]. The supernatant was diluted and used as template DNA for qPCR with primers targeting conserved bacterial regions, as stated in Table 1. Each 20 µL reaction contained 10 µL SYBR Green Master Mix (2×), 0.5 µM forward primer, 0.5 µM reverse primer, 2 µL template DNA (boiled extract), and nuclease-free water to volume. Initial denaturation at 95 °C for 5 min, followed by 40 cycles of denaturation at 95 °C for 30 s, annealing at primer-specific temperatures (48–55 °C, consistent with Table 1), and extension at 72 °C for 30 s. Melt-curve analysis was performed from 65 to 95 °C in 0.5 °C increments. 

**Table 1:** Characteristics of primers involved in qPCR quantification method

Organisms	Gene	Name	Sequence of primers$\:5{\prime\:}\to\:3{\prime\:}$	Annealing temp.	References
*P. aeruginosa*	*ecfX*	F	AGCGTTCGTCCTGCACAAGT	55 ◦C	[12]
		R	TCCACCATGCTCAGGGAGAT		
*E. coli*	*yccT*	F	GCATCGTGACCACCTTGA	51 ◦C	
		R	CAGCGTGGTGGCAAAA		
*K. pneumoniae*	*gltA*	F	ACGGCCGAATATGACGAATTC	48 ◦C	
		R	AGAGTGATCTGCTCATGAA		

Amplification was performed on a Stratagene MX3000P thermal cycler under standard conditions; species-specific annealing temperatures ($\:{55}^{\circ\:}\mathrm{C}$ for *P. aeruginosa*, $\:{51}^{\circ\:}\mathrm{C}$ for *E. coli*, and $\:{48}^{\circ\:}\mathrm{C}$ for *K. pneumoniae*) were applied. *P. aeruginosa* (EcfX, 150 bp), *E. coli* (yccT, 120 bp), *K. pneumoniae* (GltA, 180 bp). All primer pairs showed efficiencies between 90 and 105%. Standard-curve slopes ranged from − 3.2 to − 3.5, with R² ≥ 0.99. A standard curve was generated from serial 10-fold dilutions (*10⁸–10² CFU/mL*) to enable relative quantification. Standard-curve reference samples were generated from purified bacterial genomic DNA processed through the same thermal lysis and centrifugation protocol as the biofilm samples to maintain uniform matrix recovery efficiency. No-template controls (NTCs) were included in each run. Positive controls consisted of reference strains processed identically to clinical isolates. The assay reliably detected ≥ 10² CFU/mL, with quantification linearity maintained between 10²–10⁸ CFU/mL. Dilution series of template DNA were tested to confirm absence of PCR inhibition.

Linear regression was used to calculate the slope (S) of the curve, and bacterial counts were determined by comparing threshold cycle (CT) values to the standard curve. Data were normalized against control biofilms grown without antibiotics to assess modulation under sub-MIC exposure.

## Statistical analysis

This cross-sectional study analyzed 20 clinical bacterial isolates representing three species: *Pseudomonas spp.* (*n* = 5), *E. coli* (*n* = 5), and *Klebsiella spp.* (*n* = 5). Each isolate was exposed to three sub-minimum inhibitory concentration (sub-MIC) levels (12.5%, 25%, and 50%) of five antibiotics: ciprofloxacin (CIP), gentamicin (GEN), doxycycline (DOX), azithromycin (AZM), and imipenem (IMP). The primary objective was to assess differences in biofilm formation under sub-MIC exposure compared with baseline (untreated controls). For *P. aeruginosa*, *E. coli*, and *K. pneumoniae*, biofilm quantification was performed using two complementary approaches: biomass measurement via crystal violet staining and molecular assessment by qPCR.

All datasets were initially compiled in Microsoft Excel and imported into RStudio (version 2022.02.0 + 443) as CSV files for descriptive and inferential statistical analysis using the R programming language. Analyses employed the packages *tidyverse*, *rstatix*, *ggplot2*, *patchwork*, *ggsignif*, and *ggpubr*. The primary study endpoints were optical density (OD) at 620 nm (biomass method) and cycle threshold (Ct) values (qPCR), both treated as continuous variables. Each measurement was performed in triplicate for every isolate at baseline and across the three sub-MIC levels (12.5%, 25%, and 50%) of the five antibiotics. Prior to analysis, all values were normalized to the mean baseline (no antibiotic exposure).

Datasets were stratified by isolate ID and antibiotic type. Normality was assessed visually (boxplots, Q–Q plots) and statistically (Shapiro–Wilk test). When parametric assumptions were met—absence of extreme outliers, approximate normal distribution, and non-significant Mauchly’s test of sphericity—paired t-tests or one-way ANOVA were applied, followed by pairwise t-tests. For non-parametric data, Friedman tests with Dunn’s post hoc comparisons were used. Two complementary analytical approaches were undertaken: (1) paired t-tests comparing each sub-MIC category against baseline for each isolate, and (2) repeated-measures ANOVA comparing the five antibiotics within each sub-MIC level, followed by Bonferroni-adjusted pairwise tests. Finally, categorical outcomes (no effect, significant decrease, significant increase) were evaluated using Chi-square goodness-of-fit tests to determine the overall direction of biofilm modulation across species and quantification methods.

Percentages of statistically significant conditions are retained as secondary descriptive summaries after multiple-comparison correction to facilitate comparative interpretation across antibiotics. Each percentage corresponds to the fraction of isolates exhibiting a statistically significant change (increase or decrease) compared to baseline, divided by the total number of isolates tested for that antibiotic and sub-MIC level. This presentation was added to improve interpretability and allow straightforward comparison between antibiotics and concentrations.

For descriptive analysis of quantitative data, bar plots with error bars were created on summary statistics (mean ± SD) for each dataset and for each method. The bar plots are annotated with asterisks indicating the level of significance between the comparison groups. The number of asterisks indicates the significance level (*p-value < 0.05, **p-value < 0.005, ***p-value < 0.0005).

## Results

The results are presented in tables that summarize the overall significance, direction, and magnitude of biofilm responses across all tested conditions (three sub-MIC levels per antibiotic, five isolates) for comparative analysis using two complementary quantification methods—crystal violet (CV) staining and quantitative PCR (qPCR). The upper section summarizes the overall significance rates for both methods. The lower section delineates antibiotic-specific trends, expressing the magnitude and direction of each significant response across three sub-MIC concentrations and five isolates (Tables 2, 3 and 4). Detailed results are stated in the supplementary material.

**Table 2:** Comparison of the results obtained by two different biofilm evaluation methods on 5 *P. aeruginosa* isolates exposed to 3 sub-MIC concentrations each, and direction only mentioned for the significant ones

Total tested conditions = 15 (100%)	**CV** **Total significance (62%)**		**qPCR** **Total significance (47%)**		**Direction**	
	**Induction** 32% (24/75)	**Inhibition** 30% (23/75)	**Induction** 20% (15/75)	**Inhibition** 27% (20/75)	**CV**	**qPCR**
Azithromycin	93% (14/15) *		80% (12/15) *		Bidirectional	Bidirectional
	80% (12/15)	13% (2/15)	67% (10/15)	13% (2/15)	↑	↑
Ciprofloxacin	33% (5/15)		7% (1/15)			
	13% (2/15)	20% (3/15)	0% (0/15)	7% (1/15)		
Doxycycline	73% (11/15) *		73% (11/15) *		Bidirectional	Unidirectional
	67% (10/15)	7% (1/15)	0% (0/15)	73% (11/15)	↑	↓
Gentamicin	33% (5/15)		47% (7/15)			
	0% (0/15)	33% (5/15)	20% (3/15)	27% (4/15)		
Imipenem	80% (12/15) *		27% (4/15)		unidirectional	
	0% (0/15)	80% (12/15)	13% (2/15)	13% (2/15)	↓	

The asterisks indicate Total significance level, considering that all the isolates showed a statistically significant effect. Isolates that showed no change in their response are not illustrated

*p value < 0.05

### Effect of sub-MIC on *P. aeruginosa*

Total biofilm modulation significance was observed in 62% (46/75) of conditions via CV staining and 46.7% (35/75) via qPCR, reflecting substantial antibiotic-driven biofilm alterations. Azithromycin exhibited the highest overall significance in CV (93%, 14/15), followed by imipenem (80%, 12/15) and doxycycline (73%, 11/15). In qPCR, azithromycin yielded the highest significance rate (80%, 12/15), followed by doxycycline (73%, 11/15).

Across all 75 tested conditions, sub-MIC exposure produced a significant but variable inhibitory effect on *P. aeruginosa* bi (*p value < 0.05)ofilms. Overall biomass inhibition was detected slightly more frequently (30%, 23/75) than reductions in qPCR-derived genome equivalents (27%, 20/75).

Imipenem exerted the strongest inhibitory response in CV assays, significantly reducing biomass in 12/15 conditions (80%, *P* = 0.000532), compared to 2/15 (13%) in qPCR, highlighting a primary impact on extracellular matrix biomass rather than cell/DNA abundance. Conversely, doxycycline produced the greatest inhibition in qPCR, significantly reducing genome equivalents in 11/15 conditions (73%, *P* = 0.0009111), while showing minimal biomass reduction in CV (1/15, 7%). Gentamicin inhibited 5/15 conditions (33%) in CV and 4/15 (27%) in qPCR. Ciprofloxacin displayed moderate biomass inhibition (3/15, 20%) alongside minor qPCR genome equivalent reduction (1/15, 7%).

Among the 75 tested conditions, Biofilm induction was observed in 32% (24/75) of conditions by CV staining and 20% (15/75) by qPCR. Azithromycin produced the most pronounced inductive effect, enhancing biomass in 12/15 conditions (80%, *P* = 0.007526) and qPCR-derived genome equivalents in 10/15 conditions (67%). Doxycycline displayed moderate biomass induction in CV (10/15, 67%), but zero induction in qPCR (0/15, 0%).

**Fig. 1:**
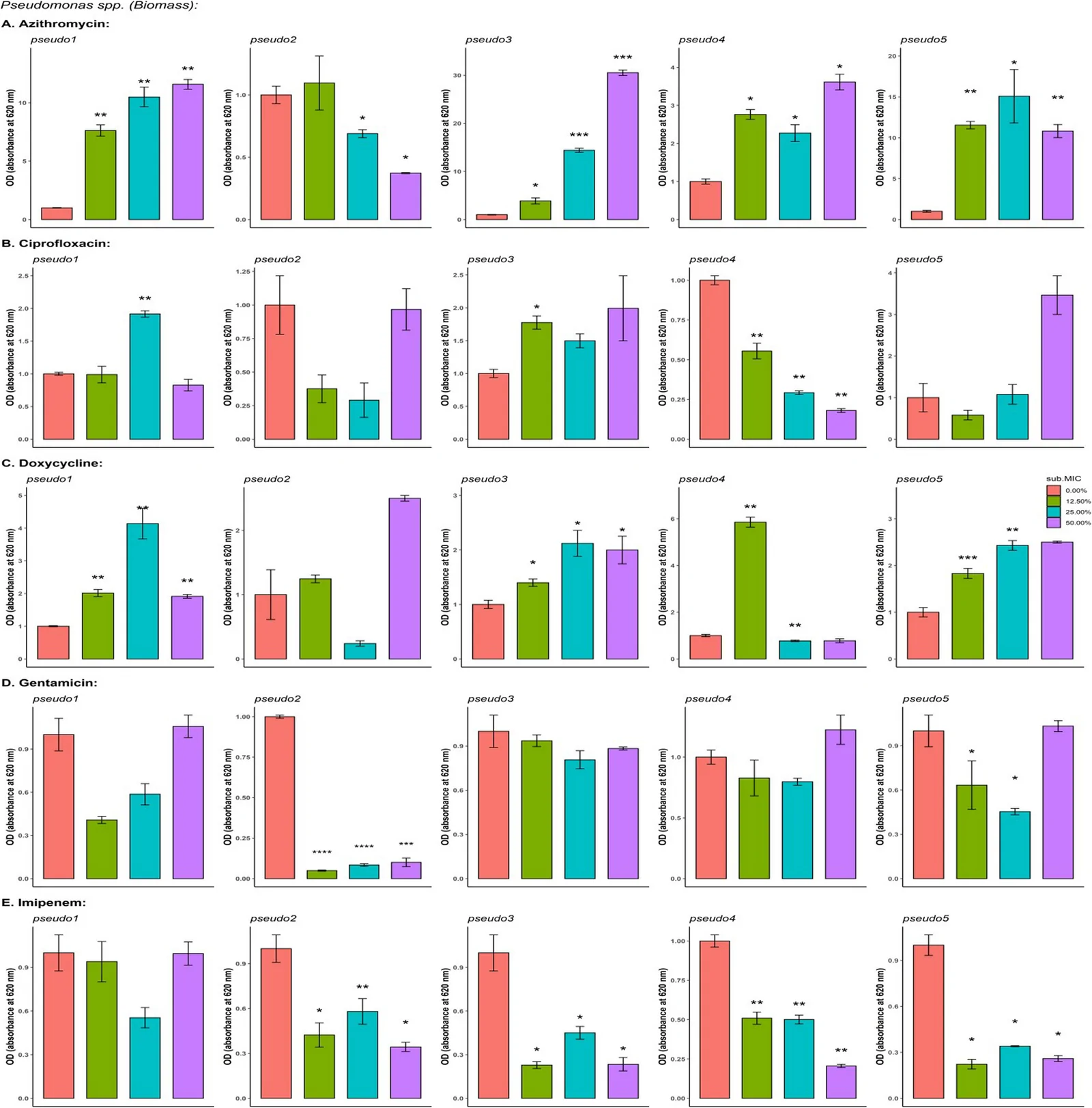
Bar plots with error bars (mean ± SD) of optical density (OD) with absorbance at 620 nm of *P. aeruginosa*. isolates (*n* = 5) by the CV staining method at baseline (no antimicrobial) and at 3 different sub-MICs (12.5%, 25%, and 50%) of AZM, CIP, DOX, GEN, and IMP, represented as panel labels (**A**, **B**, **C**, **D**, **E**), respectively. All data were normalized to their mean baseline (no antimicrobial). *Asterisks indicate the statistical significance obtained by the paired t- test. The number of asterisks indicates the significance level (*p-value < 0.05, **p-value < 0.005, ***p-value < 0.0005

**Fig. 2:**
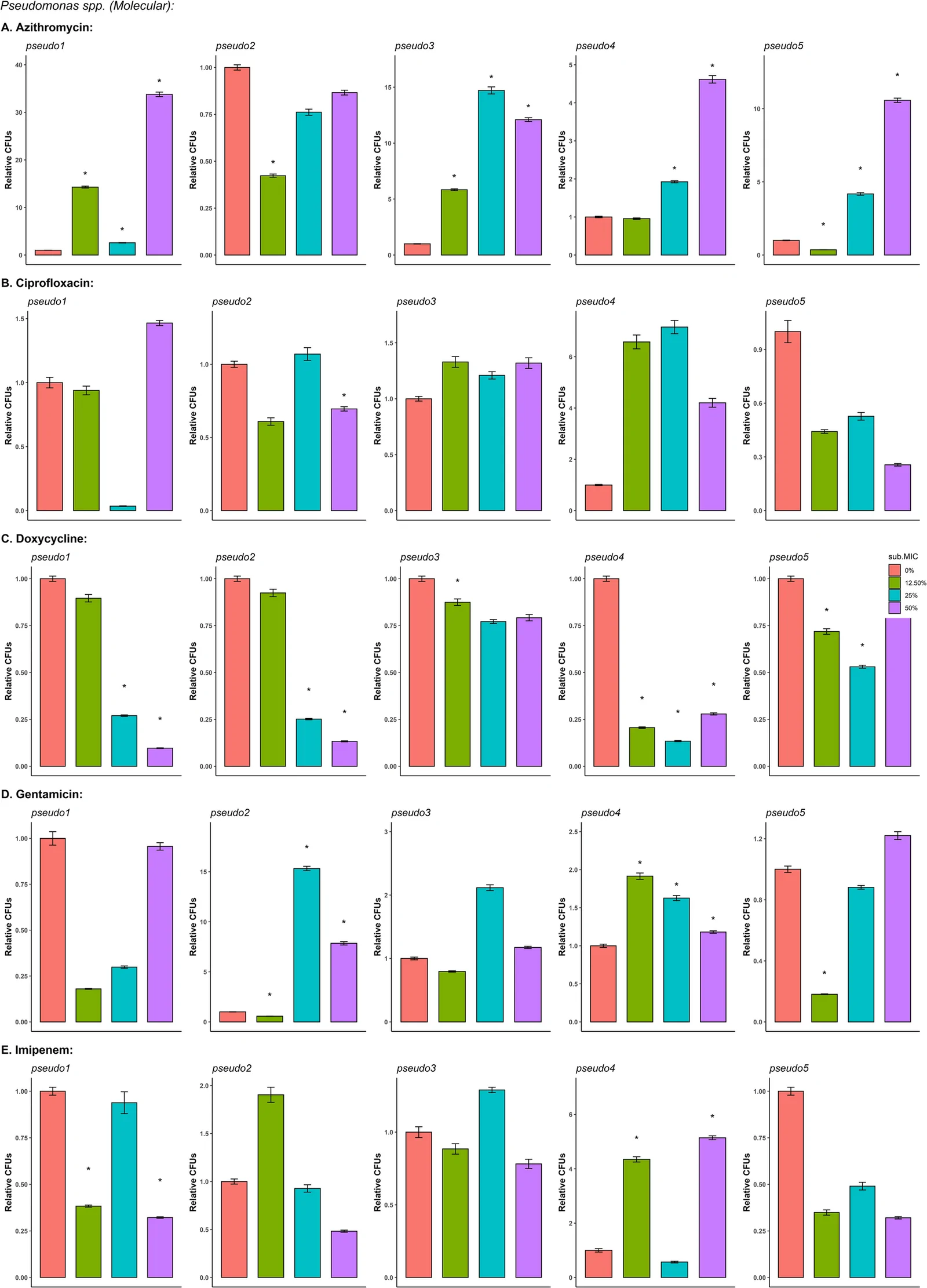
Bar plots with error bars (mean ± SD) of colony-forming units (CFU) of *P. aeruginosa*. isolates (*n* = 5) by the qPCR method at baseline (no antimicrobial) and at 3 different sub-MICs (12.5%, 25%, and 50%) of AZM, CIP, DOX, GEN, and IMP, represented as panel labels (**A**, **B**, **C**, **D**, **E**), respectively. All data were normalized to their mean baseline (no antimicrobial). *Asterisks indicate the statistical significance obtained by the paired t- test. The number of asterisks indicates the significance level (*p-value < 0.05, **p-value < 0.005, ***p-value < 0.0005

Bidirectional modulation was observed for azithromycin in both methods, as well as for doxycycline and ciprofloxacin in CV, and gentamicin and imipenem in qPCR. In contrast, unidirectional responses were observed for imipenem and gentamicin in CV (both 0% induction), and for doxycycline and ciprofloxacin in qPCR (both 0% induction), underscoring distinct, method-dependent phenotypic responses to sub-MIC antibiotic stress (Table 3; Figs. 1 and 2)

**Table 3:** Comparison of the results obtained by two different biofilm evaluation methods on 5 *E. coli* isolates exposed to 3 sub-MIC concentrations each, and direction only mentioned for the significant ones:

Total tested conditions = 15 (100%)	CV Total significance (52%)		qPCR Total significance (55%)		Direction	
	Induction 19% (14/75)	Inhibition 33% (25/75)	Induction 36% (27/75)	Inhibition 19% (14/75)	CV	qPCR
Azithromycin	73% (11/15) *		60% (9/15) *		unidirectional	Bidirectional
	0% (0/15)	73% (11/15)	53% (8/15)	7% (1/15)	↓	**↑**
Ciprofloxacin	33% (5/15)		27% (4/15)			
	0% (0/15)	33% (5/15)	0% (0/15)	27% (4/15)		
Doxycycline	27% (4/15)		67% (10/15) *			Unidirectional
	13% (2/15)	13% (2/15)	67% (10/15)	0% (0/15)		↑
Gentamicin	53% (8/15)		87% (13/15) *			bidirectional
	27% (4/15)	27% (4/15)	27% (4/15)	60% (9/15)		↓
Imipenem	73% (11/15) *		33% (5/15)		bidirectional	
	53% (8/15)	20% (3/15)	33% (5/15)	0% (0/15)	↑	

The asterisks indicate the Total significance level, considering all the isolates showed a significant effect statistically. Isolates that showed no change in their response are not illustrated

*p value < 0.05

### Effect of sub-MIC on *E. coli*

Total biofilm modulation significance was observed in 52% (39/75) of conditions via CV staining and 55% (41/75) via qPCR in *E. coli* isolates, indicating comparable overall responsiveness across both detection methods. CV assays showed a higher rate of biofilm inhibition (33%, 25/75) than induction (19%, 14/75). Conversely, qPCR revealed a higher rate of induction (36%, 27/75) than inhibition (19%, 14/75), indicating a stronger sub-MIC stimulatory effect on genome equivalents than on matrix biomass.

All tested antimicrobials demonstrated measurable biofilm inhibition, though the pattern varied substantially by method and drug class. Azithromycin exerted the strongest inhibitory effect on biomass, significantly reducing CV signals in 11 of 15 conditions (73%, *P* = 0.0009), while exhibiting predominantly inductive effects in qPCR (53%, 8/15 induction vs. 7%, 1/15 inhibition). Gentamicin showed the most pronounced inhibitory effect at the molecular level, reducing qPCR-derived genome equivalents in 9 of 15 conditions (60%, *P* = 0.0045). Overall, gentamicin influenced 87% (13/15) of qPCR conditions compared to 53% (8/15) in CV, reflecting greater molecular sensitivity. Ciprofloxacin maintained a moderate, inhibitory profile across both assays (33% CV inhibition, 27% qPCR inhibition), underscoring its role as a fluoroquinolone with reproducible biofilm-targeting activity.

**Fig. 3:**
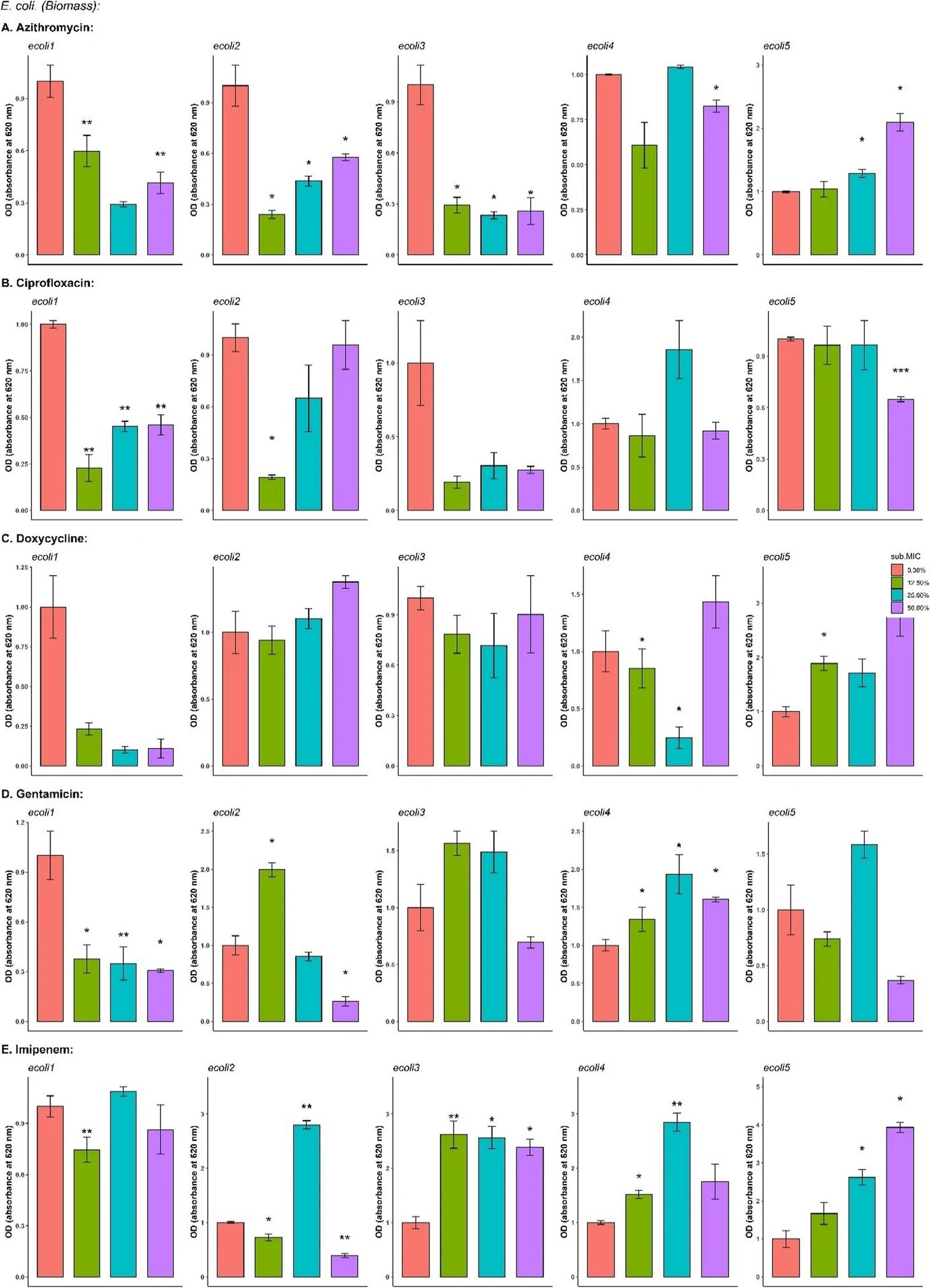
Bar plots with error bars (mean ± SD) of optical density (OD) with absorbance at 620 nm of *E. coli *isolates (*n* = 5) by crystal violet staining method at baseline (no antimicrobial) and at 3 different sub-MICs (12.5%, 25% and 50%) of AZM, CIP, DOX, GEN, and IMP, represented as panel labels (**A**, **B**, **C**, **D**, **E**), respectively. All data were normalized to their mean baseline (no antimicrobial). *Asterisks indicate the statistical significance obtained by paired t-test. The number of asterisks indicates the significance level (*p-value < 0.05, **p-value < 0.005, ***p-value < 0.0005)

**Fig. 4:**
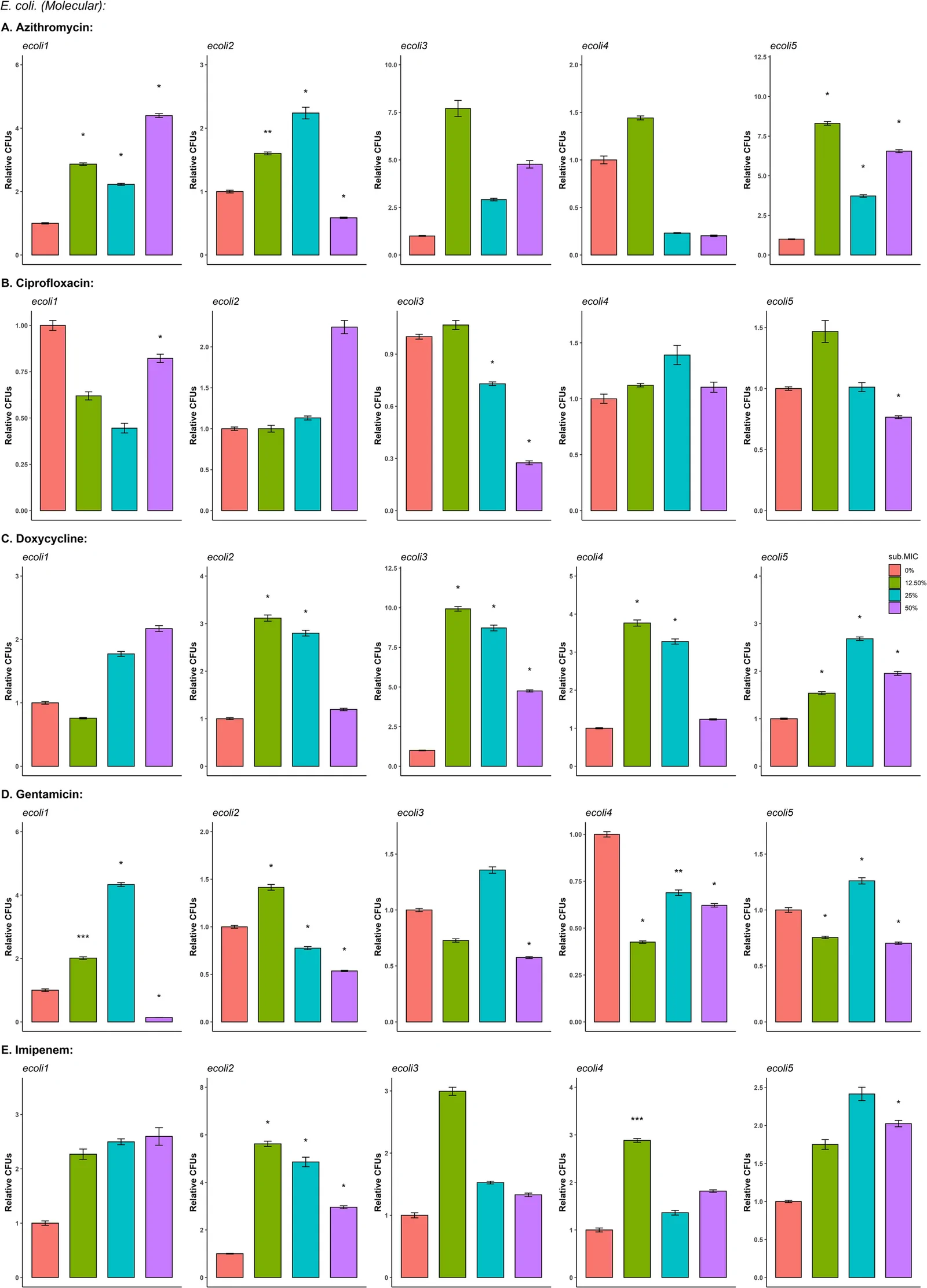
Bar plots with error bars (mean ± SD) of colony forming units (CFU) of *E. coli *isolates (*n* = 5) by qPCR method at baseline (no antimicrobial) and at 3 different sub-MICs (12.5%, 25% and 50%) of AZM, CIP, DOX, GEN and IMP, respectively, represented as panel labels (**A**, **B**, **C**, **D**, **E**). All data were normalized to their mean baseline (no antimicrobial) *Asterisks indicate the statistical significance obtained by paired t-test. The number of asterisks indicates the significance level (*p-value < 0.05, **p-value < 0.005, ***p-value < 0.0005)

Doxycycline produced the strongest unidirectional induction of qPCR-derived genome equivalents, affecting 10 of 15 conditions (67%, *P* < 0.05) with zero observed inhibition (0/15, 0%), highlighting its ability to stimulate cellular DNA accumulation without corresponding matrix biomass (13% CV induction). Imipenem demonstrated significant bidirectional modulation in CV (73%, 11/15), characterized by strong biomass induction (53%, 8/15) alongside minor inhibition (20%, 3/15), while inducing qPCR genome equivalents in 33% (5/15) of conditions (Table 4; Figs. 3 and 4).

**Table 4:** Comparison of the results obtained by two different biofilm evaluation methods on 5 *K. pneumoniae* isolates exposed to 3 sub-MIC concentrations each, and direction only mentioned for the significant ones:

Total tested conditions = 15 (100%)	**CV** **Total significance (47%)**		**qPCR** **Total significance (32%)**		**Direction**	
	**Induction** 27% (20/75)	**Inhibition** 20% (15/75)	**Induction** 21% (16/75)	**Inhibition** 11% (8/75)	**CV**	**qPCR**
Azithromycin	40% (6/15)		40% (6/15)			
	0% (0/15)	40% (6/15)	7% (1/15)	33% (5/15)		
Ciprofloxacin	60% (9/15) *		27% (4/15)		Bidirectional	
	20% (3/15)	40% (6/15)	27% (4/15)	0% (0/15)	↓	
Doxycycline	47% (7/15)		0% (0/15) *			Sig. no effect
	40% (6/15)	7% (1/15)	0% (0/15)	0% (0/15)		
Gentamicin	40% (6/15)		20% (3/15)			
	27% (4/15)	13% (2/15)	13% (2/15)	7% (1/15)		
Imipenem	47% (7/15)		60% (9/15) *			Unidirectional
	47% (7/15)	0% (0/15)	60% (9/15)	0% (0/15)		↑

The asterisks indicate Total significance level  considering all the isolates showed a significant effect statistically. Isolates showed no change in their response are not illustrated

*p value < 0.05

### Effect of sub-MIC on *Klebsiella*

Total biofilm modulation in *K. pneumoniae* was less pronounced than in *P. aeruginosa* and *E. coli*, with statistical significance observed in 47% (35/75) of conditions by CV staining and 32% (24/75) by qPCR. These findings indicate that sub-MIC antibiotic stress in *K. pneumoniae* predominantly alters total matrix biomass rather than genomic equivalents. Ciprofloxacin demonstrated the highest overall significance in CV assays (60%, 9/15), whereas imipenem displayed the highest significance in qPCR (60%, 9/15).

Among the 75 tested conditions, biofilm inhibition occurred in 20% (15/75) of cases by CV staining and 11% (8/75) by qPCR. In CV assays, all antimicrobials except imipenem (0/15, 0%) produced measurable biomass inhibition, followed by azithromycin and ciprofloxacin (both 40%, 6/15), whereas doxycycline exhibited the lowest inhibition (7%, 1/15). In qPCR, azithromycin exerted the strongest reduction in genome equivalents (33%, 5/15), followed by gentamicin (7%, 1/15), while ciprofloxacin, doxycycline, and imipenem produced no qPCR-derived inhibition (0/15, 0%).

**Fig. 5:**
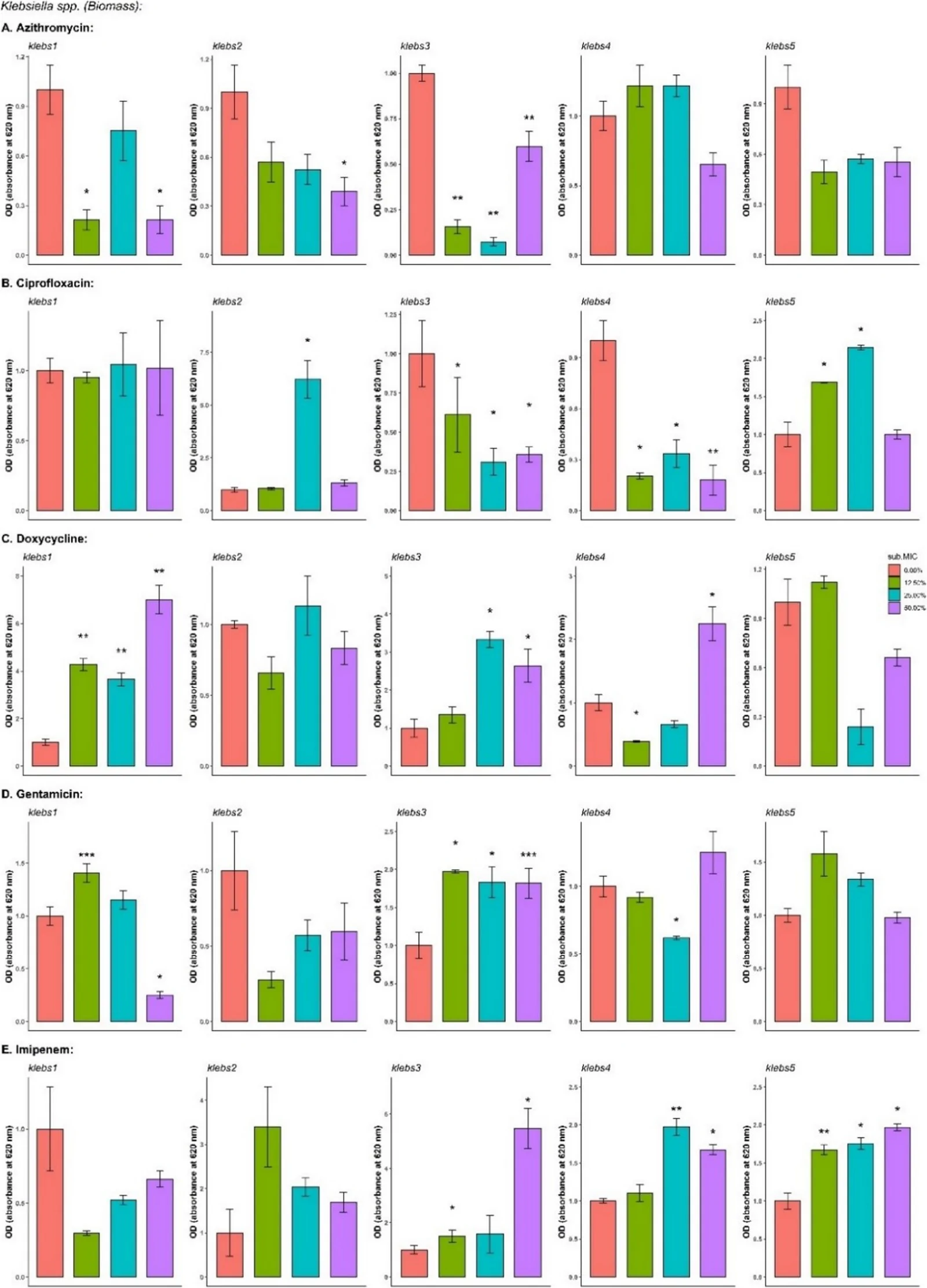
Bar plots with error bars (mean ± SD) of optical density (OD) with absorbance at 620 nm of *Klebsiella* isolates (*n* = 5) by CV staining method at baseline (no antimicrobial) and at 3 different sub-MICs (12.5%, 25% and 50%) of AZM, CIP, DOX, GEN and IMP, represented as panel labels (**A**, **B**, **C**, **D**, **E**), respectively. All data were normalized to their mean baseline (no antimicrobial). * Asterisks indicate the statistical significance obtained by paired t- test. The number of asterisks indicates the significance level (*p-value < 0.05, **p-value < 0.005, ***p-value < 0.0005)

**Fig. 6:**
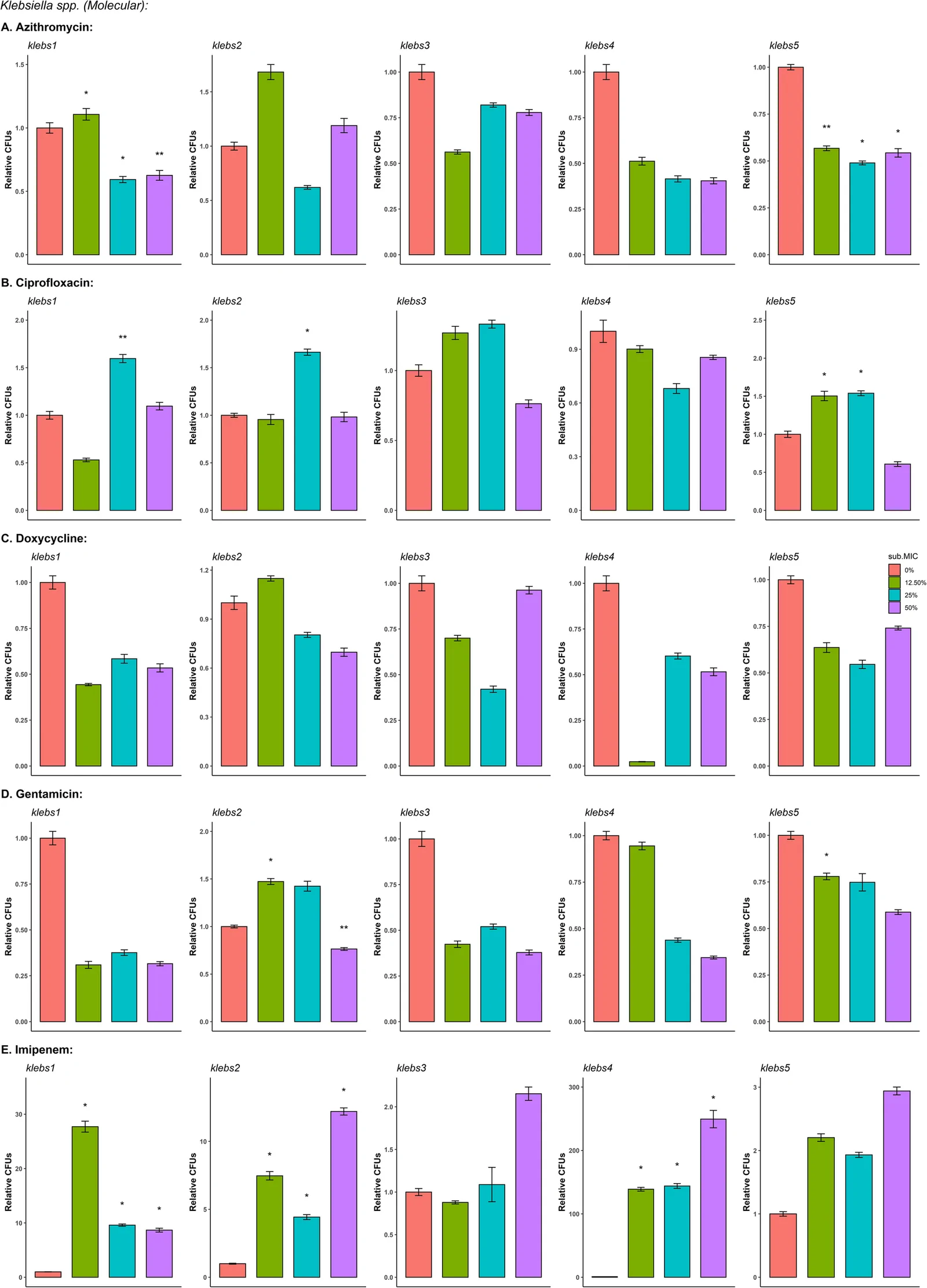
Bar plots with error bars (mean ± SD) of colony forming units (CFU) with of *Klebsiella *isolates (*n* = 5) by qPCR method at baseline (no antimicrobial) and at 3 different sub-MICs (12.5%, 25% and 50%) of AZM, CIP, DOX, GEN and IMP, represented as panel labels (**A**, **B**, **C**, **D**, **E**), respectively. All data were normalized to their mean baseline (no antimicrobial). * Asterisks indicate the statistical significance obtained by paired t- test. The number of asterisks indicates the significance level (*p-value < 0.05, **p-value < 0.005, ***p-value < 0.0005)

Biofilm induction was detected in 27% (20/75) of conditions by CV staining and 21% (16/75) by qPCR. Imipenem exhibited the strongest inductive response in both assays, enhancing qPCR genome equivalents in 60% of conditions (9/15) and matrix biomass in 47% (7/15). Doxycycline induced biomass accumulation in 40% (6/15) of conditions in CV, but yielded no effect profile in qPCR (0% induction, 0% inhibition; *P* < 0.05). Gentamicin demonstrated bidirectional modulation across both methods, inducing biomass in 27% (4/15) of CV conditions and genome equivalents in 13% (2/15) of qPCR conditions. Imipenem demonstrated unidirectional induction in qPCR, ciprofloxacin displayed bidirectional inhibition/induction in CV, and doxycycline uniquely exhibited no effect across all qPCR conditions (Table 5; Figs. 5 and 6).

**Table 5:** Standard reference strains biofilm formation Significant changes measured by Quantitative biomass method (CV staining) after exposure to 3 different sub-MICs (12.5%, 25%, and 50%) of 5 different antibiotics (azithromycin, ciprofloxacin, gentamicin, doxycycline, and imipenem) and compared to baseline by paired t-test

Isolate ID	Azithromycin	Ciprofloxacin	Doxycycline	Gentamicin	Imipenem
*pseud6* *ATCC 27,853*	25% sub-MIC; 50% sub-MIC; *p **< 0.05**	12.5% sub-MIC; 25% sub-MIC; 50% sub-MIC; ****p **< 0.00005**	12.5% sub-MIC; 25% sub-MIC; 50% sub-MIC; *p **< 0.05**	12.5% sub-MIC; 25% sub-MIC; 50% sub-MIC; **p **< 0.005**	12.5% sub-MIC; 25% sub-MIC; 50% sub-MIC; *p **< 0.05**
*klebs6* *ATCC 700,603*	12.5% sub-MIC; 25% sub-MIC; **p **< 0.005** 50% sub-MIC; ***p **< 0.0005**	N.S.	N.S.	12.5% sub-MIC; ***p **< 0.0005** 25% sub-MIC; *p **< 0.05** 50% sub-MIC; **p **< 0.005**	12.5% sub-MIC; 25% sub-MIC; 50% sub-MIC; *p **< 0.05**
*E. coli6* *ATCC 25,922*	N.S.	50% sub-MIC; *p **< 0.05**	N.S.	N.S.	N.S.

Each experiment was done in triplicates. Statistical analysis was done by paired-t test. N.S. = not significant. The number of asterisks indicates the significance level

*p-value < 0.05, **p-value <0.005, ***p-value <0.0005

### Effect of sub-MIC of antimicrobials on reference strains

**Fig. 7:**
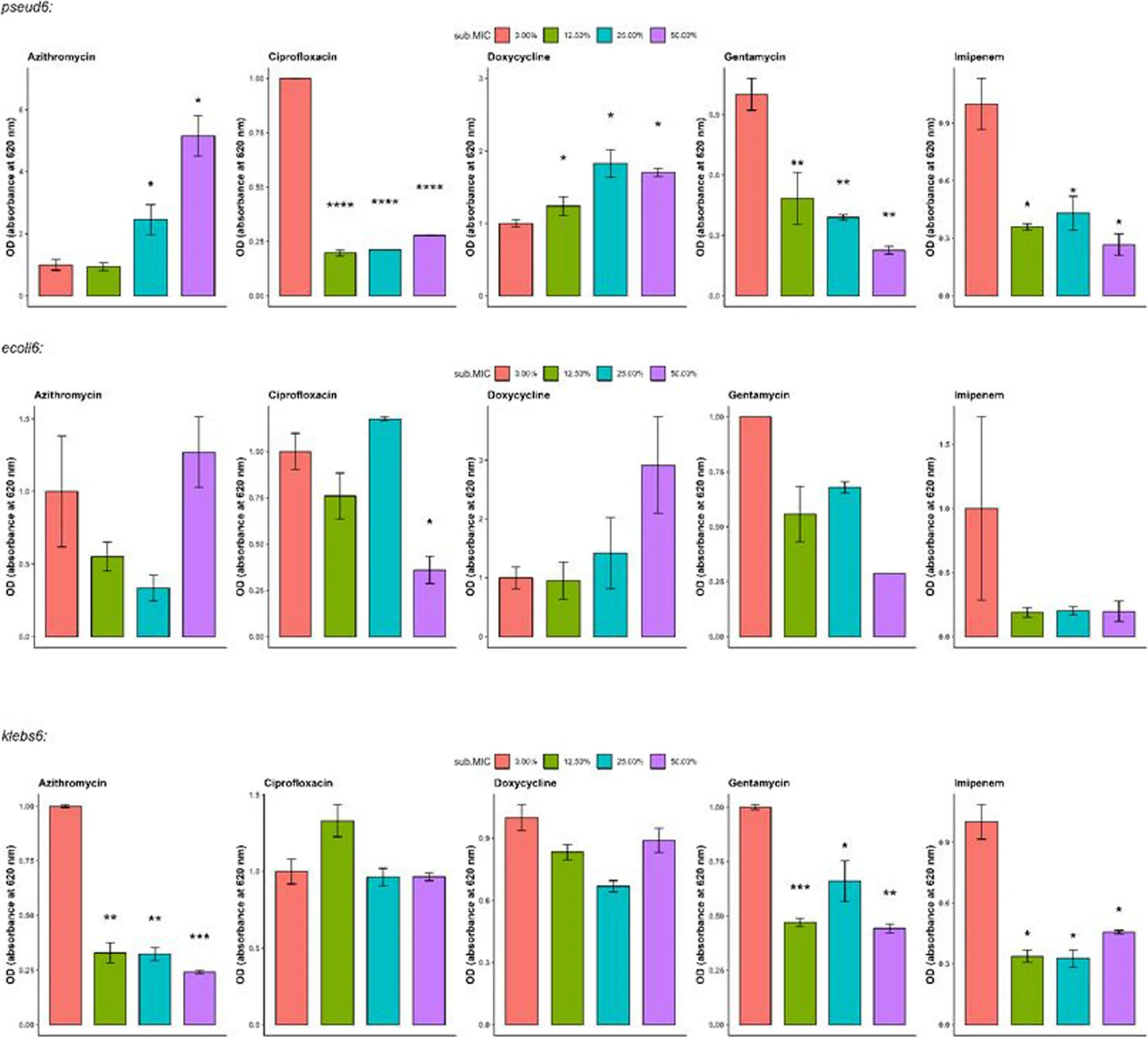
Bar plots with error bars (mean ± SD) of optical density (OD) with absorbance at 620 nm of one reference strains from each *P. aeruginosa*, *E. coli* and *K. pneumoniae* biofilm formation by CV staining method at baseline (no antimicrobial) and at 3 different sub-MICs (12.5%, 25% and 50%) of AZM, CIP, DOX, GEN and IMP, respectively. All data were normalized to their mean baseline (no antimicrobial). * Asterisks indicate the statistical significance obtained by a paired t- test. The number of asterisks indicates the significance level (*p-value < 0.05, **p-value < 0.005, ***p-value < 0.0005

*P. aeruginosa* displayed the most biofilm biomass modulation, with a 62% significance rate in CV staining. This suggests that sub-MIC exposure strongly influenced the accumulation of biofilm matrix rather than qPCR-derived genome equivalents. *E. coli* exhibited the highest bacterial growth modulation under sub-MIC exposure, with 55% significance in qPCR. This indicates that while biofilm biomass may be less affected, qPCR-derived genome equivalent within the biofilm structure was significantly altered. *K. pneumoniae* showed the lowest modulation rate in qPCR (17% significance), suggesting a weaker impact on bacterial growth. Its overall biofilm modulation was also relatively low (47% significance in CV staining), indicating that sub-MIC exposure had a less pronounced effect compared to *P. aeruginosa* and *E. coli. (*Fig. 7).

Among the tested conditions, ciprofloxacin, gentamicin, and imipenem demonstrated significant inhibitory potential across *P. aeruginosa* isolates, with imipenem showing the highest rate of biomass suppression (80% CV inhibition). In contrast, azithromycin and doxycycline predominantly stimulated biofilm biomass, consistent with their strong induction rates in CV assays (80% and 67%, respectively). Doxycycline exhibited a bidirectional effect, but its significance was skewed toward biomass induction rather than inhibition. These findings highlight that while some antibiotics consistently reduced biofilm formation, others produced variable or stimulatory responses depending on the isolate and quantification method.

In *E. coli*, only ciprofloxacin showed a significant inhibitory effect at 50% sub-MIC concentration. This was consistent with the results that showed that ciprofloxacin has only an inhibitory effect among the tested conditions.

In *K. pneumoniae*, the only antibiotics that showed a significant effect at all sub-MIC concentrations were azithromycin, gentamicin, and imipenem, that was toward decreasing the total biofilm biomass compared to the control with no antibiotic exposure. In our results, this was consistent with our results except for imipenem, which exhibited only biofilm biomass induction among the tested conditions.

### Comparison of total sub-MIC effect on the tested isolates

**Fig. 8:**
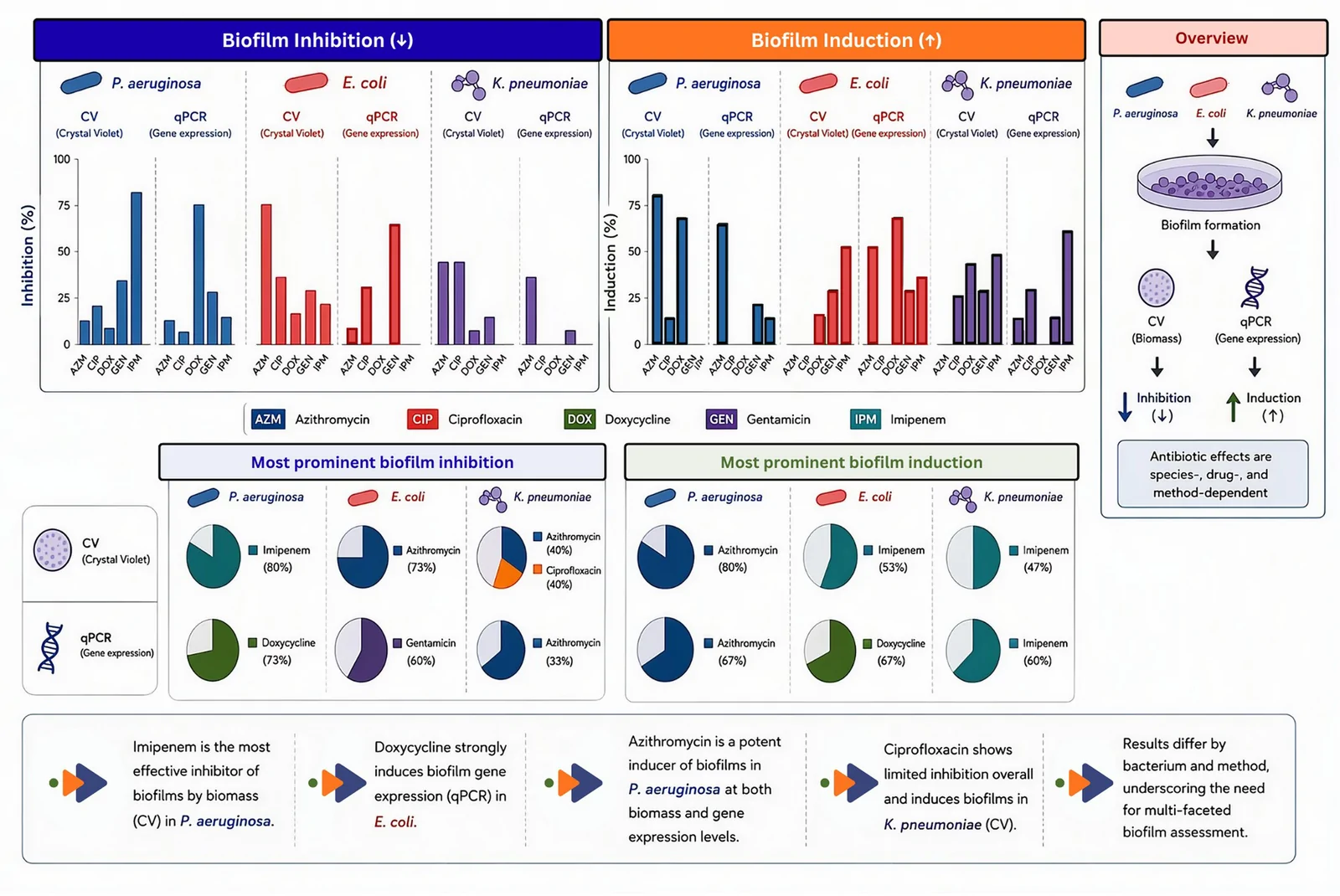
Graphical summarization for the results showing the Divergent Effects of Sub-MIC Antibiotic Exposures on Gram-Negative Biofilm Biomass and Genomic Abundance

Across all tested conditions, comparative evaluation using CV staining and qPCR revealed distinct species-dependent modulation patterns. *P. aeruginosa* exhibited the highest biofilm modulation significance in CV staining (62%), highlighting a predominant impact on matrix biomass accumulation. Conversely, *E. coli* displayed the highest qPCR-derived genome equivalent modulation rate (55%), reflecting greater sensitivity at the molecular/cellular level rather than in total biomass. *K. pneumoniae* demonstrated lower modulation, with 47% total significance in CV and 32% in qPCR (Fig. 8).

Evaluation of biofilm inhibition revealed marked strain- and isolate-specific variation across tested antimicrobials. Imipenem exerted the strongest biomass suppression in *P. aeruginosa* (80% in CV), but showed negligible qPCR inhibition in *E. coli* (0%) and *K. pneumoniae* (0%). Doxycycline-mediated genome equivalent reduction was prominent in *P. aeruginosa* (73% in qPCR), with no qPCR inhibition observed in *E. coli* (0%) and *K. pneumoniae* (0%). Azithromycin produced peak biomass inhibition in *E. coli* (73% in CV), alongside moderate inhibition in *K. pneumoniae* (40% CV, 33% qPCR) and lower activity in *P. aeruginosa* (13% CV, 13% qPCR). Ciprofloxacin exhibited consistent biomass inhibition in *K. pneumoniae* (40% CV) and *E. coli* (33% CV), but minimal to zero qPCR-derived genome equivalent suppression in *P. aeruginosa* (7% qPCR) and *K. pneumoniae* (0% qPCR). Gentamicin demonstrated strong molecular suppression in *E. coli* (60% in qPCR vs. 27% in CV), underscoring a greater impact on bacterial DNA abundance than on total matrix biomass.

Most antibiotics had an inductive effect on bacterial biomass, except imipenem on *P. aeruginosa* and both ciprofloxacin and azithromycin on *E. coli*. In the case of bacterial qPCR-derived genome equivalent reduction, Doxycycline showed no effect on *P. aeruginosa* and *K. pneumoniae*, while Ciprofloxacin did not affect *E. coli*. Azithromycin had the greatest biomass and DNA abundance, followed by Doxycycline, which showed strong biomass induction in *P. aeruginosa* and a large increase in count in *E. coli* among the other antimicrobials. Imipenem showed high induction in *K. pneumoniae* and *E. coli* but had a weaker effect on *P. aeruginosa* across both techniques. Imipenem was the strongest biofilm inhibitor in *P. aeruginosa* (80% in CV).

Sub-MIC antibiotic exposure also triggered widespread biofilm induction across all three species. Azithromycin was the primary inducer in *P. aeruginosa*, enhancing biomass in 80% of conditions (CV) and genome equivalents in 67% (qPCR), while also inducing qPCR genome equivalents in *E. coli* (53%). Doxycycline promoted strong biomass accumulation in *P. aeruginosa* (67% CV) and high genome equivalent induction in *E. coli* (67% qPCR), but no qPCR genome equivalent induction in *P. aeruginosa* (0%) and *K. pneumoniae* (0%). Imipenem induced strong responses in *K. pneumoniae* across both methods (47% CV, 60% qPCR) and in *E. coli* (53% CV, 33% qPCR), but exerted minimal inductive effect in *P. aeruginosa* (0% CV, 13% qPCR). Ciprofloxacin induced genome equivalents in *K. pneumoniae* (27% by qPCR) but showed no induction in either assay in *E. coli* (0% CV, 0% qPCR).

## Discussion

This in vitro study investigated the inhibitory potential of several antimicrobial agents at sub-MIC on biofilm formation in *P. aeruginosa*, *E. coli*, and *K. pneumoniae*. Three levels of sub-MIC exposure (12.5%, 25%, and 50%) were tested for five antimicrobial classes—ciprofloxacin (CIP), gentamicin (GEN), doxycycline (DOX), azithromycin (AZM), and imipenem (IMP)—using both CV staining to quantify total biomass and qPCR to assess bacterial cell counts within biofilms.

The sub-inhibitory antibiotic modulation of bacterial biofilms is governed by complex, species- and isolate-dependent mechanisms that diverge significantly between phenotypic biomass accumulation (CV) and molecular genome-equivalent counts (qPCR). Rather than inducing uniform inhibitory or stimulatory pathways, sub-MIC antimicrobial stress triggers distinct target-specific responses.

### Species-specific responses

Sub-MIC antibiotic stress produced distinct adaptive patterns across the three Gram-negative pathogens. *P. aeruginosa* exhibited the strongest biomass responsiveness (62% CV), reflecting matrix-directed sensitivity, with imipenem driving pronounced inhibition and azithromycin showing bidirectional induction. In contrast, *E. coli* displayed the greatest genomic modulation (55% qPCR), characterized by doxycycline-induced genomic stimulation and gentamicin-mediated suppression, underscoring its cell-level responsiveness. *K. pneumoniae* demonstrated the weakest overall modulation, with moderate biomass changes (47% CV) but minimal genomic effects (17% qPCR), responding primarily through matrix-directed alterations rather than genomic shifts. Together, these findings highlight that while *P. aeruginosa* adapts through matrix remodeling, *E. coli* responds at the genomic level, and *K. pneumoniae* remains comparatively resistant, underscoring diverse survival strategies under sub-inhibitory antibiotic exposure.

#### *P. aeruginosa*

Biofilm inhibition varied across antimicrobials. In *P. aeruginosa*, sub-MIC antibiotic exposure produced significant biofilm modulation, with alterations detected in 62% of conditions by crystal violet staining and 46.7% by qPCR. Biomass inhibition was slightly more frequent than genomic suppression, emphasizing matrix-directed sensitivity. Imipenem showed the strongest biomass inhibition (80% CV), while doxycycline primarily reduced genome equivalents (73% qPCR). Azithromycin displayed bidirectional effects, acting as both inducer and inhibitor, whereas gentamicin and ciprofloxacin produced moderate, mixed responses. These findings highlight that sub-MIC stress elicits drug-specific and method-dependent phenotypes, often decoupling biomass accumulation from genomic abundance.

*P. aeruginosa* produces three key exopolysaccharides—alginate, PEL, and PSL—whose roles in biofilm matrix stability vary by strain [13]. In parallel to our research, A study demonstrated that sub-MICs of ceftazidime significantly reduce biofilm in *P. aeruginosa* PAO1, inhibit twitching motility, and suppress bacterial adherence gene expression [14]. Similar effects of cephalosporins on biofilm and motility were noted by Roudashti et al. [7]. In contrast. Bagge et al. demonstrated that Imipenem-exposed *P. aeruginosa* biofilms display increased expression of genes coding for alginate production, resulting in thicker and more robust biofilms at sub-MIC [5].

Doxycycline sub-MIC exposure significantly induced biofilm biomass formation, also among reference strains. This aligns with evidence that *MexAB/MexXY* efflux systems in *P. aeruginosa* confer inherent resistance to tetracyclines and glycylcyclines [15, 16].Tetracycline and tigecycline sub-MIC concentrations activate the PA5471 gene, subsequently upregulating the MexXY RND efflux system, leading to enhanced biofilm survival [16, 17].

Gentamicin demonstrated consistent inhibition across both methods, though to a variable extent. Gentamicin had a significant inhibitory potential detected among 5/15 (33%) in CV and 4/15 (27%) in qPCR, although this effect was noted on reference strains. Supporting evidence shows gentamicin reduces optical density and downregulates *rh1l*, a gene linked to biofilm communication [18]. Majtan et al. reported that gentamicin inhibited alginate production at 1/4 MIC but not at 1/16 MIC, indicating dose dependency [19]. Conversely, Hoffman et al. observed a dose-dependent increase in biofilm formation with tobramycin, highlighting variability in aminoglycoside responses [20]. Strain-dependent variability was further noted, with gentamicin at 1/2 MIC promoting biofilm in some isolates while suppressing it at 1/4 MIC. These findings reinforce the strain-specific and dose-dependent nature of aminoglycoside responses. Mechanistically, oxygen limitation within biofilms alters efflux pump composition and downregulates energy metabolism genes, reducing aminoglycoside efficacy [21, 22]. Additionally, hypoxia triggers significant gene expression changes that influence antibiotic efficacy. A notable example is that the downregulation of energy metabolism genes prevents aminoglycosides from exerting their antimicrobial activity [23].

Ciprofloxacin sub-MIC exposure showed biofilm inhibition potential, even in reference strains. A study observed that ciprofloxacin reduced biofilm formation, alginate synthesis, quorum-sensing molecule production, and virulence factor secretion in *P. aeruginosa*. This effect was attributed to disrupted quorum-sensing signaling [24]. However, efflux pumps such as PA1874–1877, *MexAB-OprM*, and *MexCD-OprJ* confer resistance to fluoroquinolones, complicating therapeutic outcomes [25, 26]. Thus, while ciprofloxacin can suppress biofilm formation, efflux-mediated resistance may limit its efficacy [27].

Azithromycin exhibited strong biofilm induction in both CV and qPCR (> 80%). Despite this, azithromycin is not approved for treating *P. aeruginosa* infections, as no clinical breakpoints exist and MICs vary widely (8–512 µg/ml) [28]. Multiple studies have reported increased biofilm formation under sub-MIC macrolide exposure [20, 29]. A study found that sub-MIC clarithromycin enhanced adhesion and biofilm volume by reducing twitching motility and altering biofilm structure [30, 31]. Using methods comparable to those in our current investigation, A study [32] stated that there was growing evidence that bacteria may evolve resistance to QSIs (Azithromycin), being free to exhibit their pathogenicity after AZM concentrations. Furthermore, azithromycin may drive resistance evolution by favoring *nfxB* mutants that overproduce *MexCD-OprJ* efflux pumps [33] which, in another study, was associated with biofilm resistance to Azithromycin [27].

In contrast to our findings, A study observed that azithromycin sub-MIC reduced glycocalyx production and bacterial adherence in the *Pseudomonas* strains [34]. Similarly, J Vranes [35] suggested that subinhibitory AZM concentrations may be beneficial in preventing biofilm-associated infections [35]. Additionally, Bala et al. found that 25 clinical isolates of *P. aeruginosa* displayed decreased motility, generation of quorum-sensing signaling molecules, and biofilm formation in response to sub-MIC doses of azithromycin [36]. Collectively, these findings highlight the strain-dependent variability and mechanistic complexity of macrolide responses.

#### *E. coli*

In *E. coli*, sub-MIC antibiotics significantly modulated biofilms, with comparable responsiveness detected by crystal violet (52%) and qPCR (55%). CV assays showed more inhibition (33%) than induction (19%), while qPCR revealed stronger genomic induction (36%) than inhibition (19%). Azithromycin was the most potent biomass inhibitor (73% CV) but largely induced genome equivalents. Gentamicin exerted the strongest molecular inhibition (60% qPCR), ciprofloxacin maintained moderate inhibition across both assays, and doxycycline drove unidirectional genomic induction (67% qPCR). Imipenem displayed bidirectional biomass modulation with moderate genomic induction. These findings highlight drug-specific and method-dependent phenotypes, reinforcing the complexity of antibiotic–biofilm interactions.

Recent studies support these findings but also highlight variability. Sub-MIC exposures to ciprofloxacin, imipenem, and ceftriaxone attenuated virulence and toxin–antitoxin gene expression in *E. coli* [37]. Another study showed that sub-MIC ceftazidime, a third-generation cephalosporin, inhibited biofilm formation by interfering with bacterial cell wall synthesis and reducing motility, consistent with dose-dependent inhibitory effects reported by Sun et al. [38]. Braga et al. stated that beta-lactams, such as Cefodizime and ceftriaxone, significantly inhibited *E. coli* adhesion [39]. On the other hand, A study demonstrated that persister cell development phases influence *E. coli*’s tolerance to ampicillin, illustrating how biofilm structure can shape antibiotic response [40]. Furthermore, Sailer et al. observed that sub-MIC concentrations of amikacin and ciprofloxacin induce colanic acid production, a critical component in *E. coli* biofilm formation [41]. Adamus-Białek et al. (2019) reported that sub-MIC concentrations of amoxicillin enhance biofilm development, even in strains typically incapable of biofilm formation, emphasizing paradoxical effects of beta-lactams [42].

In this study, ciprofloxacin at 50% sub-MIC showed significant inhibitory potetial among the reference strain and also among 2 of the involved isolates when compared with the biofilm formation in the absence of ciprofloxacin. Consistent with Dong et al. [43] and D Wojnicz et al. [44] demonstrated ciprofloxacin’s ability to reduce biofilm formation, reinforcing its suppressive potential under specific conditions. Ciprofloxacin’s inhibitory effects are often attributed to disruption of quorum-sensing signaling and suppression of virulence factor secretion, though efflux pump activity can limit its efficacy. These findings highlight ciprofloxacin’s relatively consistent inhibitory profile compared to other agents.

#### *K. pneumoniae*

In *K. pneumoniae*, biofilm modulation under sub-MIC stress was less pronounced than in *P. aeruginosa* or *E. coli*, with significance observed in 47% of conditions by crystal violet and 32% by qPCR. Overall, biomass changes were more frequent than genomic alterations. Ciprofloxacin showed the highest CV significance (60%), while imipenem was most significant in qPCR (60%). Biofilm inhibition was limited (20% CV, 11% qPCR), with azithromycin and ciprofloxacin reducing biomass moderately, and azithromycin exerting the strongest genomic inhibition. Induction was more common (27% CV, 21% qPCR), driven largely by imipenem, which enhanced both biomass and genome equivalents. Doxycycline induced biomass but had no qPCR effect, while gentamicin displayed bidirectional modulation. These findings highlight that *K. pneumoniae* biofilms respond to sub-MIC stress primarily through matrix-directed changes, with weaker genomic sensitivity compared to other species.

Several studies have examined the impact of antimicrobials on multidrug-resistant (MDR) and extensively drug-resistant (XDR) *K. pneumoniae* biofilms. A study confirmed our results with doxycycline, stating that sub-MIC tetracyclines enhance biofilm formation in minocycline-resistant *K. pneumoniae* via increased surface hydrophobicity and eDNA, reduced membrane permeability, and abnormal cell elongation [45]. Anderl et al. (2000) observed that the CFU count in the biofilms after 24 h of antimicrobial exposure did not differ from the count after 4 h of treatment. It revealed that wild-type *K. pneumoniae* biofilms were impermeable to ampicillin but penetrated by ciprofloxacin, whereas a β-lactamase-deficient mutant permitted ampicillin penetration. Despite these differences, ampicillin and ciprofloxacin failed to eradicate wild-type *K. pneumoniae* biofilms, reinforcing the protective role of biofilm architecture [46]. Furthermore, a study on urinary strains reported a positive correlation between biofilm-forming capacity and antimicrobial resistance profiles in XDR *K. pneumoniae* [47]. However, data on *Klebsiella* biofilm modulation under sub-MIC exposure remain limited, highlighting a critical gap for further investigation.

### Differential antibiotic-associated matrix remodeling vs. genomic abundance alteration

Several adaptive mechanisms may explain the observed heterogeneity. At sub-MIC levels, antibiotics act less as bactericidal agents and more as signaling cues, triggering drug-specific cellular programs that reinforce biofilm resilience.

Sub-MIC antibiotic stress revealed clear mechanistic divergence. Cell wall and protein synthesis inhibitors such as imipenem in *P. aeruginosa* (80% CV vs. 13% qPCR inhibition) and azithromycin in *E. coli* (73% CV vs. 7% qPCR inhibition) primarily suppressed extracellular matrix formation while sparing genomic equivalents. In contrast, translation and replication inhibitors like doxycycline in *P. aeruginosa* (73% qPCR vs. 7% CV inhibition) and gentamicin in *E. coli* (60% qPCR vs. 27% CV inhibition) predominantly reduced genomic replication with minimal matrix impact. Bidirectional phenotypes were common: azithromycin induced biofilm in *P. aeruginosa* but inhibited biomass in *E. coli*, while imipenem acted as a strong biomass inhibitor in *P. aeruginosa* yet shifted to an inductive role in *K. pneumoniae*. These findings underscore that sub-MIC stress selectively uncouples matrix remodeling from genomic replication, producing species- and drug-specific adaptive outcomes. Meanwhile, sub-MIC exposures frequently display non-linear dose-response patterns. Such non-monotonic behavior underscores that antibiotics at sub-MIC levels act primarily as signaling molecules.

A reduction in biofilm matrix alongside an increase in DNA abundance may reflect a shift toward a dispersal-prone or planktonic phenotype under sub-MIC antibiotic stress. This response may be driven by the selective downregulation of matrix biosynthesis genes (e.g., *psl*, *pel*, *algD* in *P. aeruginosa*), weakening the extracellular polymeric substance (EPS) scaffold while permitting continued bacterial proliferation [14]. Another possibility is metabolic uncoupling, where sub-MIC exposure redirects energy from matrix production toward cell division [48]. QS interference is also a plausible contributor; they may inhibit matrix gene expression while allowing solitary cell proliferation [30]. Also, sub-MIC exposures may induce matrix-degrading enzymes like DNases, proteases, or polysaccharide lyases, reducing matrix density while leaving viable cells intact or even enhanced [26]. These mechanisms highlight how sub-MIC stress can either reinforce biofilm survival through matrix expansion or promote dispersal and planktonic growth, underscoring the complexity of bacterial adaptation under sublethal antimicrobial pressure.

The presence of negatively charged extracellular polysaccharides prevents the penetration of oppositely charged antimicrobials such as tobramycin, as well as uncharged ones like β-lactams, thereby contributing to resistance gene expression within the biofilm [49]. Additionally, eDNA creates a cation-limited environment within the biofilm, further protecting bacteria from antimicrobial action. In *P. aeruginosa*, eDNA induces lipopolysaccharide (LPS) operon modifications, enhancing resistance mechanisms [50]. Due to its cation-chelating properties, eDNA reduces the effectiveness of positively charged antimicrobials [50]. This eDNA acts as an electrostatic scaffold that stabilizes biofilm architecture and chelates cations, providing localized protection against antimicrobials even when replication is suppressed. Although ciprofloxacin, an uncharged quinolone, has been shown to penetrate biofilms efficiently, its efficacy can be compromised by excessive polysaccharide production. This suggests that biofilm overproduction not only obstructs antimicrobial penetration but also alters bacterial physiology by restricting access to nutrients and oxygen [2]. Moreover, exposure to sub-MIC of antimicrobials induces mutagenesis, further increasing bacterial resistance. In *P. aeruginosa*, for instance, sub-MIC exposure to ciprofloxacin leads to resistance development via *gyrA* and *gyrB* gene mutations [51]. This underscores how sub-MIC may drive adaptive resistance pathways.

Some investigations report induction of biofilm formation, while others demonstrate inhibition. Even without bactericidal activity, sub-MIC levels are capable of altering bacterial morphology, pathogenicity, and biofilm dynamics [6, 7]. Clinically, this is alarming: nearly half of nosocomial infections are associated with indwelling medical devices, and most bacterial infections in humans are linked to biofilms. Conventional antimicrobials are increasingly ineffective against biofilm-related infections [52]. Sub-MIC exposure is not a rare event—it occurs at the beginning and end of treatment regimens, between dosing intervals, and during prolonged low-dose therapies. These conditions create fertile ground for mutagenesis, resistance selection, and the onset of biofilm development [53]. Within biofilms, the risks are amplified: the dense extracellular matrix and close microbial proximity provide ideal conditions for plasmid-mediated resistance transfer. Moreover, quorum-sensing networks orchestrate collective bacterial behavior, enabling microorganisms to withstand antimicrobial pressure and evade host immune defenses [48].

### Paradigm shift in biofilm diagnostics: dual-target methodological considerations

Our findings reveal a critical blind spot in standard biofilm methodology: single-assay evaluations—most notably the widespread reliance on CV staining alone—are fundamentally inadequate for assessing sub-inhibitory antimicrobial activity. Relying solely on biomass staining risks misinterpreting matrix collapse or dispersal as bacterial eradication, while genomically intact sessile populations may persist and continue driving infection. Conversely, relying only on genomic quantification risks missing drug‑induced extracellular matrix expansion, which can hinder immune clearance and limit antibiotic penetration in vivo.

Our dual-method approach—CV staining for biomass and qPCR for bacterial biofilm DNA abundance—proved essential for capturing the complexity of biofilm modulation. CV staining alone has been criticized for its inability to assess synergism or antagonism in multispecies biofilms, such as those associated with bacterial vaginosis [54]. Another recent study compared CV staining with microscopy and qPCR to assess biofilm dynamics. It found that CV alone may not fully capture biofilm population dynamics [55, 56].

Discrepancies between the two methods confirm that matrix suppression does not necessarily equate to biofilm-associated DNA abundance. Biofilm-associated DNA exists both as genomic material within viable sessile cells and as structural eDNA secreted or released via prophage-mediated autolysis [57]. Sub-MIC concentrations of DNA-damaging or protein-synthesis-inhibiting agents, such as fluoroquinolones and aminoglycosides, can shift the balance between intact cells and structural eDNA. qPCR primarily reflected cellular abundance and genomic integrity, whereas CV staining bound non-specifically to the entire polyanionic matrix—including eDNA, exopolysaccharides, and proteins—highlighting the importance of multimodal quantification. The divergence between CV and qPCR outcomes may pinpoint two distinct adaptive phenotypes: survival-oriented matrix upregulation, where replication is suppressed but matrix reinforced, and dispersal-prone phenotypes, where matrix reduction accompanies maintained or elevated cell counts [55].

This methodological insight is critical, as reliance on a single assay may lead to misinterpretation of biofilm dynamics. By employing both CV and qPCR, our study highlights the necessity of multimodal quantification to assess biofilm responses accurately under antimicrobial pressure and directly addresses concerns about assay limitations.

### Limitation

While this study provides comprehensive baseline insights into sub-MIC biofilm dynamics, several methodological constraints qualify its clinical generalizability. First, static in vitro microtiter assays lack the fluid shear, nutrient flux, and physiological gradients (e.g., host proteins, immune effectors, oxygenation) characteristic of clinical infection sites and indwelling devices. Second, our monomicrobial framework omits interspecies interactions that frequently govern polymicrobial bio-architectures. Third, qPCR quantifies total genome equivalents—encompassing viable, dead, lysed, and eDNA—rather than active cellular viability; direct physiological viability requires CFU enumeration or PMA-qPCR validation.

These findings highlight the intricate, isolate-dependent risks of sub-inhibitory drug exposure. Future investigations utilizing dynamic flow cells, polymicrobial models, and in vivo systems are required to validate these phenotypes and map underlying mechanisms, including efflux pump upregulation, quorum-sensing crosstalk, and temporal eDNA dynamics.

## Conclusion

This study highlights the complex and species-specific interactions between sub-MIC of antibiotics and biofilm formation in *P. aeruginosa*, *E. coli*, and *K. pneumoniae*. The results demonstrate that sub-MIC exposure can exert both inhibitory and stimulatory effects, varying by bacterial species, antibiotic class, and assessment method. While azithromycin and gentamicin showed consistent biofilm suppression across all isolates, other antibiotics displayed bidirectional modulation, highlighting the complexity of antibiotic-biofilm interactions. The discrepancy between crystal violet staining and qPCR findings reinforces the necessity of employing multi-method evaluations to accurately assess biofilm dynamics. Biofilm biomass inhibition was observed more frequently than bacterial count reduction, suggesting that sub-MIC exposure primarily alters matrix composition rather than directly eliminating bacterial cells. Additionally, extracellular polymeric substances, efflux pump modifications, and metabolic adaptations contribute to biofilm resilience under sub-MIC conditions. These findings offer valuable insights into potential biofilm-targeted interventions using sub-MIC antibiotics, emphasizing the need for species-specific antimicrobial strategies. Future investigations should focus on unraveling the molecular pathways underlying these responses and optimizing treatment approaches for biofilm-associated infections.

## Supplementary Information

Supplementary Material 1.

## Data Availability

All data supporting the findings of this study are available within the paper and its Supplementary Information.
