# Supplementary material for: Beyond lethal doses: sub-MIC levels of multi-class antibiotics induce species- and isolate-dependent biofilm inhibition in gram-negative pathogens
Source: BMC Microbiol. 2026 Sep 26;26:838. doi: 10.1186/s12866-026-05620-5 (PMC13615587; doi:10.1186/s12866-026-05620-5)
Supplement: Supplementary file 1 [file 12866_2026_5620_MOESM1_ESM.docx]

**Table S 1 A table including the strains' names, sources, collecting sites, and MIC:**

| *Isolate* | Specimen Source | Infection Type | Isolate ID | MIC (µg/mL) CIP | MIC GEN | MIC DOX | MIC AZM | MIC IMP |
| --- | --- | --- | --- | --- | --- | --- | --- | --- |
| *P. aeruginosa 1* | sputum | Cystic fibrosis | ***pseudo1*** | 0.25 | 2 | 4 | 64 | 1 |
| *P. aeruginosa 2* | sputum | Cystic fibrosis | ***pseudo2*** | 0.5 | 2 | 4 | 128 | 1 |
| *P. aeruginosa 3* | sputum | Cystic fibrosis | ***pseudo3*** | 0.25 | 1 | 2 | 32 | 0.5 |
| *P. aeruginosa 4* | Respiratory aspirate | Pneumonia | ***pseudo4*** | 0.5 | 2 | 4 | >128 | 1 |
| *P. aeruginosa 5* | Respiratory aspirate | Pneumonia | ***pseudo5*** | 0.25 | 2 | 4 | 64 | 1 |
| *E. coli 1* | Urine | UTI | ***ecoli1*** | 0.25 | 1 | 2 | 64 | 0.25 |
| *E. coli 2* | Urine | UTI | ***ecoli2*** | 0.5 | 2 | 2 | 64 | 0.5 |
| *E. coli 3* | Urine | UTI | ***ecoli3*** | 0.25 | 1 | 4 | >128 | 0.25 |
| *E. coli 4* | Urine | UTI | ***ecoli4*** | 0.25 | 2 | 2 | 64 | 0.25 |
| *E. coli 5* | Urine | UTI | ***ecoli5*** | 0.5 | 2 | 4 | 64 | 0.5 |
| *K. pneumoniae 1* | Respiratory aspirate | Pneumonia | ***kleb1*** | 0.25 | 1 | 2 | 64 | 0.25 |
| *K. pneumoniae 2* | Blood culture | Septicemia | ***kleb2*** | 0.5 | 2 | 2 | 64 | 0.5 |
| *K. pneumoniae 3* | CRBSI | IV catheter | ***kleb3*** | 0.25 | 1 | 4 | >128 | 0.25 |
| *K. pneumoniae 4* | Respiratory aspirate | pneumonia | ***kleb4*** | 0.25 | 2 | 2 | 64 | 0.25 |
| *K. pneumoniae 5* | Respiratory aspirate | pneumonia | ***kleb5*** | 0.5 | 2 | 4 | >128 | 0.5 |

(CRBSIs); Central-venous-catheter-related bloodstream infections

**Table S 2** : Quantitation of biofilm formation by biomass method (crystal violet staining) of *Pseudomonas* spp. isolates exposed to 3 different sub-MICs (12.5%, 25%, and 50%) of 5 different antibiotics (azithromycin, ciprofloxacin, gentamicin, doxycycline, and imipenem) and compared to baseline by paired t-test.

| **Isolate ID** | **Azithromycin** | **Ciprofloxacin** | **Doxycycline** | **Gentamicin** | **Imipenem** |
| --- | --- | --- | --- | --- | --- |
| ***pseud1*** | 12.5% sub-MIC;  25% sub-MIC;  50% sub-MIC;  *****p* <0.005** | 25% sub-MIC;  *****p* <0.005** | 12.5% sub-MIC;  25% sub-MIC;  50% sub-MIC;  *****p* <0.005** | N.S. | N.S. |
| ***pseud2*** | 25% sub-MIC;  50% sub-MIC;  ****p* <0.05** | N.S. | N.S. | 12.5% sub-MIC;  25% sub-MIC;  *******p* <0.0005**  50% sub-MIC;  ******p* <0.0005** | 12.5% sub-MIC;  ****p* <0.05**  25% sub-MIC;  *****p* <0.005**  50% sub-MIC;  ****p* <0.05** |
| ***pseud3*** | 12.5% sub-MIC  ****p* <0.05**  25% sub-MIC  50% sub-MIC  ******p* <0.0005** | 12.5% sub-MIC;  ****p* <0.05** | 12.5% sub-MIC;  25% sub-MIC;  50% sub-MIC;  ****p* <0.05** | N.S. | 12.5% sub-MIC;  25% sub-MIC;  50% sub-MIC;  ****p* <0.05** |
| ***pseud4*** | 12.5% sub-MIC;  25% sub-MIC;  50% sub-MIC;  ****p* <0.05** | 12.5% sub-MIC;  25% sub-MIC;  50% sub-MIC;  *****p* <0.005** | 12.5% sub-MIC;  25% sub-MIC;  *****p* <0.005** | N.S. | 12.5% sub-MIC;  25% sub-MIC;  50% sub-MIC;  *****p* <0.005** |
| ***pseud5*** | 12.5% sub-MIC;  *****p* <0.005**  25% sub-MIC;  ****p* <0.05**  50% sub-MIC;  *****p* <0.005** | N.S. | 12.5% sub-MIC;  ******p* <0.0005**  25% sub-MIC;  50% sub-MIC;  *****p* <0.005** | 12.5% sub-MIC;  25% sub-MIC;  ****p* <0.05** | 12.5% sub-MIC;  25% sub-MIC;  50% sub-MIC;  ****p* <0.05** |

Each experiment was done in triplicates. Statistical analysis was done by paired-t test. N.S. = not significant. The number of asterisks indicates the significance level (**p*-value < 0.05, ***p*-value <0.005, ****p*-value <0.0005).

Table S 3 : Summary of significant sub-MICs compared to baseline of Pseudomonas spp. isolates by biomass method.

| *Pseudomonas* spp.  (n=5) | **Azithromycin** | **Ciprofloxacin** | **Doxycycline** | **Gentamicin** | **Imipenem** |
| --- | --- | --- | --- | --- | --- |
| 12.5% sub-MIC | 4/5 (80%) | 2/5 (40%) | 4/5 (80%) | 2/5 (40%) | 4/5 (80%) |
| 25% sub-MIC | 5/5 (100%) | 2/5 (40%) | 4/5 (80%) | 2/5 (40%) | 4/5 (80%) |
| 50% sub-MIC | 5/5 (100%) | 1/5 (20%) | 3/5 (60%) | 1/5 (20%) | 4/5 (80%) |
| Total | **14/15 (93%)** | 5/15 (33%) | 11/15 (73%) | 5/15 (33%) | **12/15 (80%)** |

Table S 4: Summary of direction of significance of sub-MICs of Pseudomonas spp. isolates by biomass method.

| **Azithromycin** | Significant increase | Significant decrease | Not significant | Total |
| --- | --- | --- | --- | --- |
| 12.5% sub-MIC | 4 | 0 | 1 | 5 |
| 25% sub-MIC | 4 | 1 | 0 | 5 |
| 50% sub-MIC | 4 | 1 | 0 | 5 |
| **Total** | **12** | **2** | **1** | **15** |
| **Ciprofloxacin** | | | |  |
| 12.5% sub-MIC | 1 | 1 | 3 | 5 |
| 25% sub-MIC | 1 | 1 | 3 | 5 |
| 50% sub-MIC | 0 | 1 | 4 | 5 |
| **Total** | **2** | **3** | **10** | **15** |
| **Doxycycline** | | | |  |
| 12.5% sub-MIC | 4 | 0 | 1 | 5 |
| 25% sub-MIC | 3 | 1 | 1 | 5 |
| 50% sub-MIC | 3 | 0 | 2 | 5 |
| **Total** | **10** | **1** | **4** | **15** |
| **Gentamicin** | | | |  |
| 12.5% sub-MIC | 0 | 2 | 3 | 5 |
| 25% sub-MIC | 0 | 2 | 3 | 5 |
| 50% sub-MIC | 0 | 1 | 4 | 5 |
| **Total** | **0** | **5** | **10** | **15** |
| **Imipenem** | | | |  |
| 12.5% sub-MIC | 0 | 4 | 1 | 5 |
| 25% sub-MIC | 0 | 4 | 1 | 5 |
| 50% sub-MIC | 0 | 4 | 1 | 5 |
| **Total** | **0** | **12** | **3** | **15** |

Table S 5: The direction of the significant effect on biofilm formation of azithromycin and imipenem sub-MICs in Pseudomonas spp. isolates

| ***Pseudomonas spp.*** | sig. decrease | sig. increase | total | X^2^ | dof | *p-*value |
| --- | --- | --- | --- | --- | --- | --- |
| AZM | 2 | 12 | **14** | 7.1429 | 1 | **0.007526** |
| DOX | 1 | 10 | 11 | 7.3636 | 1 | **0.006656** |
| IMP | 12 | 0 | **12** | 12 | 1 | **0.000532** |

N.S., not significant. dof, degree of freedom. AZM; azithromycin, IMP; imipenem. *P-*values are obtained by Chi square goodness of fit test. *P-* values at <0.05 are

Table S 6: Quantitative PCR of Pseudomonas spp. isolates exposed to 3 different sub-MICs (12.5%, 25%, and 50%) of 5 different antibiotics (azithromycin, ciprofloxacin, gentamicin, doxycycline, and imipenem) and compared to baseline by paired t-test

| **Isolate  ID** | **Azithromycin** | **Ciprofloxacin** | **Doxycycline** | **Gentamicin** | **Imipenem** |
| --- | --- | --- | --- | --- | --- |
| ***pseud1*** | 12.5% sub-MIC  25% sub-MIC;  50% sub-MIC;  ****p* <0.05** | N.S. | 25% sub-MIC;  50% sub-MIC;  ****p* <0.05** | N.S. | 12.5% sub-MIC  50% sub-MIC;  ****p* <0.05** |
| ***pseud2*** | 12.5% sub-MIC  ****p* <0.05** | 50% sub-MIC;  ****p* <0.05** | 25% sub-MIC;  50% sub-MIC;  ****p* <0.05** | 12.5% sub-MIC  25% sub-MIC;  50% sub-MIC;  ****p* <0.05** | N.S. |
| ***pseud3*** | 12.5% sub-MIC  25% sub-MIC;  50% sub-MIC;  ****p* <0.05** | N.S. | 12.5% sub-MIC;  ****p* <0.05** | N.S. | N.S. |
| ***pseud4*** | 25% sub-MIC;  50% sub-MIC;  ****p* <0.05** | N.S. | 12.5% sub-MIC  25% sub-MIC;  50% sub-MIC;  ****p* <0.05** | 12.5% sub-MIC  25% sub-MIC;  50% sub-MIC;  ****p* <0.05** | 12.5% sub-MIC;  50% sub-MIC;  ****p* <0.05** |
| ***pseud5*** | 12.5% sub-MIC  25% sub-MIC;  50% sub-MIC;  ****p* <0.05** | N.S. | 12.5% sub-MIC  25% sub-MIC;  50% sub-MIC;  ****p* <0.05** | 12.5% sub-MIC;  ****p* <0.05** | N.S. |

Each experiment was done in triplicates. Statistical analysis was done by paired-t test. N.S. = not significant. The number of asterisks indicates the significance level (**p*-value < 0.05, ***p*-value <0.005, ****p*-value <0.0005).

Table S 7: Summary of significant sub-MIC categories compared to baseline in Pseudomonas spp. isolates by qPCR.

| *Pseudomonas* spp.  (n=5) | **Azithromycin** | **Ciprofloxacin** | **Doxycycline** | **Gentamicin** | **Imipenem** |
| --- | --- | --- | --- | --- | --- |
| 12.5% sub-MIC | 4/5 (80%) | 0/5 (0%) | 3/5 (60%) | 3/5 (60%) | 2/5 (40%) |
| 25% sub-MIC | 4/5 (80%) | 0/5 (0%) | 4/5 (80%) | 2/5 (40%) | 0/5 (0%) |
| 50% sub-MIC | 4/5 (80%) | 1/5 (20%) | 4/5 (80%) | 2/5 (40%) | 2/5 (40%) |
| Total | 12/15 (80%) | 1/15 (6.7%) | 11/15 (73%) | 7/15 (47%) | 4/15 (27%) |

Table S 8: Summary of direction of significance of sub-MICs of *Pseudomonas* spp. isolates by qPCR.

| **Azithromycin** | Significant increase | Significant decrease | Not significant | Total |
| --- | --- | --- | --- | --- |
| 12.5% sub-MIC | 2 | 2 | 1 | 5 |
| 25% sub-MIC | 4 | 0 | 1 | 5 |
| 50% sub-MIC | 4 | 0 | 1 | 5 |
| **Total** | **10** | **2** | **3** | **15** |
| **Ciprofloxacin** | | | |  |
| 12.5% sub-MIC | 0 | 0 | 5 | 5 |
| 25% sub-MIC | 0 | 0 | 5 | 5 |
| 50% sub-MIC | 0 | 1 | 4 | 5 |
| **Total** | **0** | **1** | **14** | **15** |
| **Doxycycline** | | | |  |
| 12.5% sub-MIC | 0 | 3 | 2 | 5 |
| 25% sub-MIC | 0 | 4 | 1 | 5 |
| 50% sub-MIC | 0 | 4 | 1 | 5 |
| **Total** | **0** | **11** | **4** | **15** |
| **Gentamicin** | | | |  |
| 12.5% sub-MIC | 1 | 2 | 2 | 5 |
| 25% sub-MIC | 2 | 0 | 3 | 5 |
| 50% sub-MIC | 0 | 2 | 3 | 5 |
| **Total** | **3** | **4** | **8** | **15** |
| **Imipenem** | | | |  |
| 12.5% sub-MIC | 1 | 1 | 3 | 5 |
| 25% sub-MIC | 0 | 0 | 5 | 5 |
| 50% sub-MIC | 1 | 1 | 3 | 5 |
| **Total** | **2** | **2** | **11** | **15** |

Table S 9: The direction of the significant effect on biofilm formation of azithromycin, doxycycline and gentamicin sub-MICs in Pseudomonas spp. isolates by qPCR.

| ***Pseudomonas spp*.** | sig. decrease | sig. increase | Total | X^2^ | df | *p-*value |
| --- | --- | --- | --- | --- | --- | --- |
| **AZM** | **2** | **10** | **12** | **5.3333** | **1** | **0.02092** |
| **DOX** | **11** | **0** | **11** | **11** | **1** | **0.0009111** |
| **Gen** | **4** | **3** | **7** | **0.066667** | **1** | **0.007963** |

Table S 10: Quantitation of biofilm formation by biomass method (crystal violet staining) of E. coli. isolates exposed to 3 different sub-MIC (12.5%, 25%, and 50%) of 5 different antibiotics (azithromycin, ciprofloxacin, gentamicin, doxycycline, and imipenem) and compared to baseline by paired t-test.

| **Isolate  ID** | **Azithromycin** | **Ciprofloxacin** | **Doxycycline** | **Gentamicin** | **Imipenem** |
| --- | --- | --- | --- | --- | --- |
| ***ecoli1*** | 25% sub-MIC;  50% sub-MIC;  *****p* <0.005** | 12.5% sub-MIC;  25% sub-MIC;  50% sub-MIC;  *****p* <0.005** | N.S. | 12.5% sub-MIC;  ****p* <0.05**  25% sub-MIC;  *****p* <0.005**  50% sub-MIC;  ****p* <0.05** | 12.5% sub-MIC;  *****p* <0.005** |
| ***ecoli2*** | 12.5% sub-MIC;  25% sub-MIC;  50% sub-MIC;  ****p* <0.05** | 12.5% sub-MIC;  ****p* <0.05** | N.S. | 12.5% sub-MIC;  50% sub-MIC;  ****p* <0.05** | 12.5% sub-MIC;  ****p* <0.05**  25% sub-MIC;  50% sub-MIC;  *****p* <0.005** |
| ***ecoli3*** | 12.5% sub-MIC;  25% sub-MIC;  50% sub-MIC;  ****p* <0.05** | N.S. | N.S. | N.S. | 12.5% sub-MIC;  *****p* <0.005**  25% sub-MIC;  50% sub-MIC;  ****p* <0.05** |
| ***ecoli4*** | 50% sub-MIC;  ****p* <0.05** | N.S. | 12.5% sub-MIC;  25% sub-MIC;  ****p* <0.05** | 12.5% sub-MIC;  25% sub-MIC;  50% sub-MIC;  ****p* <0.05** | 12.5% sub-MIC;  ****p* <0.05**  25% sub-MIC;  *****p* <0.005** |
| ***ecoli5*** | 25% sub-MIC;  50% sub-MIC;  ****p* <0.05** | 50% sub-MIC;  ******p* <0.0005** | 12.5% sub-MIC;  50% sub-MIC;  ****p* <0.05** | N.S. | 25% sub-MIC;  50% sub-MIC;  ****p* <0.05** |

Each experiment was done in triplicates. Statistical analysis was done by paired-t test. N.S. = not significant. The number of asterisks indicates the significance level (**p*-value < 0.05, ***p*-value <0.005, ****p*-value <0.000

Table S 11: Summary of significant sub-MICs compared to baseline of E. coli isolates by biomass method

| *E. coli* spp.  (n=5) | **Azithromycin** | **Ciprofloxacin** | **Doxycycline** | **Gentamicin** | **Imipenem** |  |
| --- | --- | --- | --- | --- | --- | --- |
| 12.5% sub-MIC | 3/5 (60%) | 2/5 (40%)33333 | 2/5 (40%) | 3/5 (60%) | 4/5 (80%) |  |
| 25% sub-MIC | 3/5 (60%) | 1/5 (20%) | 1/5 (20%) | 2/5 (40%) | 4/5 (80%) |  |
| 50% sub-MIC | 5/5 (100%) | 2/5 (40%) | 1/5 (20%) | 3/5 (60%) | 3/5 (60%) |  |
| Total | **11/15 (73%)** | 5/15 (33%) | 4/15 (27%) | 8/15 (53%) | **11/15 (73%)** |  |

Table S 12: Summary of direction of significance of sub-MICs of *E. coli* isolates by biomass method.

| **Azithromycin** | Significant increase | Significant decrease | Not significant | total |
| --- | --- | --- | --- | --- |
| 12.5% sub-MIC | 0 | 3 | 2 | 5 |
| 25% sub-MIC | 0 | 3 | 2 | 5 |
| 50% sub-MIC | 0 | 5 | 0 | 5 |
| **Total** | **0** | **11** | **4** | **15** |
| **Ciprofloxacin** | | | |  |
| 12.5% sub-MIC | 0 | 2 | 3 | 5 |
| 25% sub-MIC | 0 | 1 | 4 | 5 |
| 50% sub-MIC | 0 | 2 | 3 | 5 |
| **Total** | **0** | **5** | **10** | **15** |
| **Doxycycline** | | | |  |
| 12.5% sub-MIC | 1 | 1 | 3 | 5 |
| 25% sub-MIC | 0 | 1 | 4 | 5 |
| 50% sub-MIC | 1 | 0 | 4 | 5 |
| **Total** | **2** | **2** | **11** | **15** |
| **Gentamicin** | | | |  |
| 12.5% sub-MIC | 2 | 1 | 2 | 5 |
| 25% sub-MIC | 1 | 1 | 3 | 5 |
| 50% sub-MIC | 1 | 2 | 2 | 5 |
| **Total** | **4** | **4** | **7** | **15** |
| **Imipenem** | | | |  |
| 12.5% sub-MIC | 2 | 2 | 1 | 5 |
| 25% sub-MIC | 4 | 0 | 1 | 5 |
| 50% sub-MIC | 2 | 1 | 2 | 5 |
| **Total** | **8** | **3** | **4** | **15** |

Table S 13: The direction of the significant effect on biofilm formation of azithromycin and imipenem sub-MICs in E. coli isolates.

| ***E. coli* isolates** | sig. decrease | sig. increase | total | X^2^ | dof | *p-*value |
| --- | --- | --- | --- | --- | --- | --- |
| AZM | 11 | 0 | 11 | 11 | 1 | **0.0009111** |
| IMP | 3 | 8 | 11 | 2.2727 | 1 | 0.001317 |

Table S 14: Quantitative PCR of E. coli. isolates exposed to 3 different sub-MICs (12.5%, 25%, and 50%) of 5 different antibiotics (azithromycin, ciprofloxacin, gentamicin, doxycycline, and imipenem) and compared to baseline by paired t-test.

| **Isolate  ID** | **Azithromycin** | **Ciprofloxacin** | **Doxycycline** | **Gentamicin** | **Imipenem** |
| --- | --- | --- | --- | --- | --- |
| ***ecoli1*** | 12.5% sub-MIC;  25% sub-MIC;  50% sub-MIC;  ****p* <0.05** | 50% sub-MIC;  ****p* <0.05** | N.S. | 12.5% sub-MIC;  ******p* <0.0005**  25% sub-MIC;  50% sub-MIC;  ****p* <0.05** | N.S. |
| ***ecoli2*** | 12.5% sub-MIC;  *****p* <0.005**  25% sub-MIC;  50% sub-MIC;  ****p* <0.05** | N.S. | 12.5% sub-MIC;  25% sub-MIC;  ****p* <0.05** | 12.5% sub-MIC;  25% sub-MIC;  50% sub-MIC;  ****p* <0.05** | 12.5% sub-MIC;  25% sub-MIC;  50% sub-MIC;  ****p* <0.05** |
| ***ecoli3*** | N.S. | 25% sub-MIC;  50% sub-MIC;  ****p* <0.05** | 12.5% sub-MIC;  25% sub-MIC;  50% sub-MIC;  ****p* <0.05** | 50% sub-MIC;  ****p* <0.05** | N.S. |
| ***ecoli4*** | N.S. | N.S. | 12.5% sub-MIC;  25% sub-MIC;  ****p* <0.05** | 12.5% sub-MIC  ****p* <0.05**  25% sub-MIC;  *****p* <0.005**  50% sub-MIC;  ****p* <0.05** | 12.5% sub-MIC;  ******p* <0.0005** |
| ***ecoli5*** | 12.5% sub-MIC;  25% sub-MIC;  50% sub-MIC;  ****p* <0.05** | 50% sub-MIC;  ****p* <0.05** | 12.5% sub-MIC;  25% sub-MIC;  50% sub-MIC;  ****p* <0.05** | 12.5% sub-MIC;  25% sub-MIC;  50% sub-MIC;  ****p* <0.05** | 50% sub-MIC;  ****p* <0.05** |

Each experiment was done in triplicates. Statistical analysis was done by paired-t test. N.S. = not significant. The number of asterisks indicates the significance level (**p*-value < 0.05, ***p*-value <0.005, ****p*-value <0.0005).

Table S 15: Summary of significant sub-MICs compared to baseline in E. coli isolates by qPCR.

| *E. coli* spp.  (n=5) | **Azithromycin** | **Ciprofloxacin** | **Doxycycline** | **Gentamicin** | **Imipenem** |
| --- | --- | --- | --- | --- | --- |
| 12.5% sub-MIC | 3/5 (60%) | 0/5 (0%) | 4/5 (80%) | 4/5 (80%) | 2/5 (40%) |
| 25% sub-MIC | 3/5 (60%) | 1/5 (20%) | 4/5 (80%) | 4/5 (80%) | 1/5 (20%) |
| 50% sub-MIC | 3/5 (60%) | 3/5 (60%) | 2/5 (40%) | 5/5 (100%) | 2/5 (40%) |
| Total | 9/15 (60%) | 4/15 (27%) | 10/15 (67%) | **13/15 (80%)** | 5/15 (33%) |

Table S 16: Summary of direction of significance of sub-MICs of E. coli isolates by qPCR.

| **Azithromycin** | Significant increase | Significant decrease | Not significant | total |
| --- | --- | --- | --- | --- |
| 12.5% sub-MIC | 3 | 0 | 2 | 5 |
| 25% sub-MIC | 3 | 0 | 2 | 5 |
| 50% sub-MIC | 2 | 1 | 2 | 5 |
| **Total** | **8** | **1** | **6** | **15** |
| **Ciprofloxacin** | | | |  |
| 12.5% sub-MIC | 0 | 0 | 5 | 5 |
| 25% sub-MIC | 0 | 1 | 4 | 5 |
| 50% sub-MIC | 0 | 3 | 2 | 5 |
| **Total** | **0** | **4** | **11** | **15** |
| **Doxycycline** | | | |  |
| 12.5% sub-MIC | 4 | 0 | 1 | 5 |
| 25% sub-MIC | 4 | 0 | 1 | 5 |
| 50% sub-MIC | 2 | 0 | 3 | 5 |
| **Total** | **10** | **0** | **5** | **15** |
| **Gentamicin** | | | |  |
| 12.5% sub-MIC | 2 | 2 | 1 | 5 |
| 25% sub-MIC | 2 | 2 | 1 | 5 |
| 50% sub-MIC | 0 | 5 | 0 | 5 |
| **Total** | **4** | **9** | **2** | **15** |
| **Imipenem** | | | |  |
| 12.5% sub-MIC | 2 | 0 | 3 | 5 |
| 25% sub-MIC | 1 | 0 | 4 | 5 |
| 50% sub-MIC | 2 | 0 | 3 | 5 |
| **Total** | **5** | **0** | **10** | **15** |

Table S 17: The direction of the significant effect on biofilm formation of azithromycin and doxycycline sub-MICs in E. coli. Isolates by qPCR.

| ***E. coli* isolates** | sig. decrease | sig. increase | Total | X^2^ | dof | *p-*value |
| --- | --- | --- | --- | --- | --- | --- |
| GEN | 9 | 4 | 13 | 1.9231 | 1 | 0.001655 |

Table S 18: Quantitation of biofilm formation by biomass method (crystal violet staining) of Klebsiella spp. isolates exposed to 3 different sub-MIC (12.5%, 25%, and 50%) of 5 different antibiotics (azithromycin, ciprofloxacin, gentamicin, doxycycline, and imipenem) and compared to baseline by paired t-test.

| **Isolate ID** | **Azithromycin** | **Ciprofloxacin** | **Doxycycline** | **Gentamicin** | **Imipenem** |
| --- | --- | --- | --- | --- | --- |
| ***klebs1*** | 12.5% sub-MIC  50% sub-MIC  ****p* <0.05** | N.S. | 12.5% sub-MIC  25% sub-MIC  50% sub-MIC  *****p* <0.005** | 12.5% sub-MIC  ******p* <0.0005**  50% sub-MIC  ****p* <0.05** | N.S. |
| ***klebs2*** | 50% sub-MIC  ****p* <0.05** | 25% sub-MIC  ****p* <0.05** | N.S. | N.S. | N.S. |
| ***klebs3*** | 12.5% sub-MIC  25% sub-MIC  50% sub-MIC  *****p* <0.005** | 12.5% sub-MIC  25% sub-MIC  50% sub-MIC  ****p* <0.05** | 25% sub-MIC  50% sub-MIC  ****p* <0.05** | 12.5% sub-MIC  25% sub-MIC  ****p* <0.05**  50% sub-MIC  ******p* <0.0005** | 12.5% sub-MIC  50% sub-MIC  ****p* <0.05** |
| ***klebs4*** | N.S. | 12.5% sub-MIC  25% sub-MIC  ****p* <0.05**  50% sub-MIC  *****p* <0.005** | 12.5% sub-MIC  50% sub-MIC  ****p* <0.05** | 25% sub-MIC  ****p* <0.05** | 25% sub-MIC  *****p* <0.005**  50% sub-MIC  ****p* <0.05** |
| ***klebs5*** | N.S. | 12.5% sub-MIC  25% sub-MIC  ****p* <0.05** | N.S. | N.S. | 12.5% sub-MIC  *****p* <0.005**  25% sub-MIC  50% sub-MIC  ****p* <0.05** |

Each experiment was done in triplicates. Statistical analysis was done by paired-t test. N.S. = not significant. The number of asterisks indicates the significance level (**p*-value < 0.05, ***p*-value <0.005, ****p*-value <0.0005).

**Table S 19: Summary of significant sub-MICs compared to baseline of *Klebsiella* spp. isolates by biomass method.**

| *Klebsiella* spp. (n=5) | **Azithromycin** | **Ciprofloxacin** | **Doxycycline** | **Gentamicin** | **Imipenem** |
| --- | --- | --- | --- | --- | --- |
| 12.5% sub-MIC | 2/5 (40%) | 3/5 (60%) | 2/5 (40%) | 2/5 (40%) | 2/5 (40%) |
| 25% sub-MIC | 1/5 (20%) | 4/5 (80%) | 2/5 (40%) | 2/5 (40%) | 2/5 (40%) |
| 50% sub-MIC | 3/5 (60%) | 2/5 (40%) | 3/5 (60%) | 2/5 (40%) | 3/5 (60%) |
| Total | 6/15 (40%) | **9/15 (60%)** | 7/15 (47%) | 6/15 (40%) | 7/15 (47%) |

Table S 20: Summary of direction of significance of sub-MICs of *Klebsiella spp.* isolates by biomass method.

| **Azithromycin** | Significant increase | Significant decrease | Not significant | total |
| --- | --- | --- | --- | --- |
| 12.5% sub-MIC | 0 | 2 | 3 | 5 |
| 25% sub-MIC | 0 | 1 | 4 | 5 |
| 50% sub-MIC | 0 | 3 | 2 | 5 |
| **Total** | **0** | **6** | **9** | **15** |
| **Ciprofloxacin** | | | |  |
| 12.5% sub-MIC | 1 | 2 | 2 | 5 |
| 25% sub-MIC | 2 | 2 | 1 | 5 |
| 50% sub-MIC | 0 | 2 | 3 | 5 |
| **Total** | **3** | **6** | **6** | **15** |
| **Doxycycline** | | | |  |
| 12.5% sub-MIC | 1 | 1 | 3 | 5 |
| 25% sub-MIC | 2 | 0 | 3 | 5 |
| 50% sub-MIC | 3 | 0 | 2 | 5 |
| **Total** | **6** | **1** | **8** | **15** |
| **Gentamicin** | | | |  |
| 12.5% sub-MIC | 2 | 0 | 3 | 5 |
| 25% sub-MIC | 1 | 1 | 3 | 5 |
| 50% sub-MIC | 1 | 1 | 3 | 5 |
| **Total** | **4** | **2** | **9** | **15** |
| **Imipenem** | | | |  |
| 12.5% sub-MIC | 2 | 0 | 3 | 5 |
| 25% sub-MIC | 2 | 0 | 3 | 5 |
| 50% sub-MIC | 3 | 0 | 2 | 5 |
| **Total** | **7** | **0** | **8** | **15** |

Table S 21: Quantitative PCR of Klebsiella spp. isolates exposed to 3 different sub-MICs (12.5%, 25%, and 50%) of 5 different antibiotics (azithromycin, ciprofloxacin, gentamicin, doxycycline, and imipenem) and compared to baseline by paired t-test.

| **Isolate  ID** | **Azithromycin** | **Ciprofloxacin** | **Doxycycline** | **Gentamicin** | **Imipenem** |
| --- | --- | --- | --- | --- | --- |
| ***klebs1*** | 12.5% sub-MIC;  25% sub-MIC;  ****p* <0.05**  50% sub-MIC;  *****p* <0.005** | 25% sub-MIC;  *****p* <0.005** | N.S. | N.S. | 12.5% sub-MIC;  25% sub-MIC;  50% sub-MIC;  ****p* <0.05** |
| ***klebs2*** | N.S. | 25% sub-MIC;  ****p* <0.05** | N.S. | 12.5% sub-MIC  ****p* <0.05**  50% sub-MIC;  *****p* <0.005** | 12.5% sub-MIC;  25% sub-MIC;  50% sub-MIC;  ****p* <0.05** |
| ***klebs3*** | N.S. | N.S. | N.S. | N.S. | N.S. |
| ***klebs4*** | N.S. | N.S. | N.S. | N.S. | 12.5% sub-MIC;  25% sub-MIC;  50% sub-MIC;  ****p* <0.05** |
| ***klebs5*** | 12.5% sub-MIC;  *****p* <0.005**  25% sub-MIC;  50% sub-MIC;  ****p* <0.05** | 12.5% sub-MIC  25% sub-MIC;  ****p* <0.05** | N.S. | 12.5% sub-MIC;  ****p* <0.05** | N.S. |

Each experiment was done in triplicates. Statistical analysis was done by paired-t test. N.S. = not significant. The number of asterisks indicates the significance level (**p*-value < 0.05, ***p*-value <0.005, ****p*-value <0.0005).

Table S 22: Summary of significant sub-MIC categories compared to baseline in *Klebsiella spp.* isolates by qPCR.

| *Klebsiella* spp.  (n=5) | **Azithromycin** | **Ciprofloxacin** | **Doxycycline** | **Gentamicin** | **Imipenem** |
| --- | --- | --- | --- | --- | --- |
| 12.5% sub-MIC | 2/5 (40%) | 1/5 (20%) | 0/5 (0%) | 2/5 (40%) | 3/5 (60%) |
| 25% sub-MIC | 2/5 (40%) | 3/5 (60%) | 0/5 (0%) | 0/5 (0%) | 3/5 (60%) |
| 50% sub-MIC | 2/5 (40%) | 0/5 (0%) | 0/5 (0%) | 1/5 (20%) | 3/5 (60%) |
| Total | 6/15 (40%) | 4/15 (27%) | 0/15 (0%) | 3/15 (20%) | **9/15 (60%)** |

Table S 23: Summary of direction of significance of sub-MICs of Klebsiella spp. isolates by qPCR

| **Azithromycin** | Significant increase | Significant decrease | Not significant | total |
| --- | --- | --- | --- | --- |
| 12.5% sub-MIC | 1 | 1 | 3 | 5 |
| 25% sub-MIC | 0 | 2 | 3 | 5 |
| 50% sub-MIC | 0 | 2 | 3 | 5 |
| **Total** | **1** | **5** | **9** | **15** |
| **Ciprofloxacin** | | | |  |
| 12.5% sub-MIC | 1 | 0 | 4 | 5 |
| 25% sub-MIC | 3 | 0 | 2 | 5 |
| 50% sub-MIC | 0 | 0 | 5 | 5 |
| **Total** | **4** | **0** | **11** | **15** |
| **Doxycycline** | | | |  |
| 12.5% sub-MIC | 0 | 0 | 5 | 5 |
| 25% sub-MIC | 0 | 0 | 5 | 5 |
| 50% sub-MIC | 0 | 0 | 5 | 5 |
| **Total** | **0** | **0** | **15** | **15** |
| **Gentamicin** | | | |  |
| 12.5% sub-MIC | 1 | 1 | 3 | 5 |
| 25% sub-MIC | 0 | 0 | 5 | 5 |
| 50% sub-MIC | 1 | 0 | 4 | 5 |
| **total** | **2** | **1** | **12** | **15** |
| **Imipenem** | | | |  |
| 12.5% sub-MIC | 3 | 0 | 2 | 5 |
| 25% sub-MIC | 3 | 0 | 2 | 5 |
| 50% sub-MIC | 3 | 0 | 2 | 5 |
| **total** | **9** | **0** | **6** | **15** |
